# Peripheral-Central Neuroimmune Crosstalk in Parkinson's Disease: What Do Patients and Animal Models Tell Us?

**DOI:** 10.3389/fneur.2019.00232

**Published:** 2019-03-19

**Authors:** Marie Therese Fuzzati-Armentero, Silvia Cerri, Fabio Blandini

**Affiliations:** Laboratory of Cellular and Molecular Neurobiology, IRCCS Mondino Foundation, Pavia, Italy

**Keywords:** inflammasomes, cytokine, toxin-induced models, LRRK2, lymphocytes, blood-brain barrier, MPTP, 6-OHDA

## Abstract

The brain is no longer considered an immune privileged organ and neuroinflammation has long been associated with Parkinson's disease. Accumulating evidence demonstrates that innate and adaptive responses take place in the CNS. The extent to which peripheral immune alterations impacts on the CNS, or vice and versa, is, however, still a matter of debate. Gaining a better knowledge of the molecular and cellular immune dysfunctions present in these two compartments and clarifying their mutual interactions is a fundamental step in understanding and preventing Parkinson's disease (PD) pathogenesis. This review provides an overview of the current knowledge on inflammatory processes evidenced both in PD patients and in toxin-induced animal models of the disease. It discusses differences and similarities between human and animal studies in the context of neuroinflammation and immune responses and how they have guided therapeutic strategies to slow down disease progression. Future longitudinal studies are necessary and can help gain a better understanding on peripheral-central nervous system crosstalk to improve therapeutic strategies for PD.

## Introduction

Innate and adaptive immunity are crucial for the survival of all organisms (1). The innate system as we know it today is the result of a long evolutionary period. Innate immunity promotes inflammation as an immediate, non-specific response to infection, insults and/or biological stressors through a limited set of germline-encoded receptors expressed on specialized cells: macrophages, dendritic cells, natural killer cells, or neutrophils (2). While it does not provide long-lasting immunity it is pivotal to the overall immune response. Adaptive immunity possibly developed and evolved as a complementary plug-in system to strengthen innate immunity in complex organisms (3). Adaptive immunity requires activation of the innate system and subsequent antigen presentation to adaptive immune cells and is based on unlimited somatic diversification of receptors present on lymphoid cells and their selective expansion to match pathogens (4). The adaptive immunity repertoire is specific for each individual, shaped by individual life history, and is the foundation of a strong memory response that allows rapid reaction to repeated infections. Combination of innate and adaptive immunity is required for the comprehensive immune protection observed in humans.

The capacities of the central nervous system (CNS), recalling those provided by the adaptive immune system, evolves in response to each individual's life experience. In the timeline of evolution, the CNS has developed amidst the innate and adaptive immune systems, combining characteristics to allow the appearance and development of increasingly more complex organisms. Throughout evolution the CNS and the two arms of the immune system have co-evolved through constant crosstalk and communication, persistently improving their ability to respond and adapt to the environment. Today, the brain is no longer considered an immune privileged organ. Innate and adaptive responses take place in the CNS (5), and peripheral immune alterations can impact on the CNS. Gaining a better knowledge of the molecular and cellular immune dysfunctions present in these two compartments, clarifying and understanding their mutual interactions represents a fundamental step in the development of alternative therapeutic strategies for neurodegenerative diseases, including Parkinson's disease (PD). PD is not considered an immune disease, but it is now widely accepted that inflammation and neuroinflammation are important players in the etiology and/or the progression of the disease; the triad, inflammation, neuroinflammation, and neurodegeneration, likely intervening in a vicious, each one sustaining the other. A possible role of viral infection in PD etiology has been extensively addressed but is still a matter of debate (6).

Here we will review some inflammatory processes that take place both in the central and peripheral compartments. In particular, we will look at communication barriers that limit passage of information, as well as the regulation of inflammatory markers and cells that may transit from the periphery to the brain, and vice e versa. We will summarize data on the inflammasome, an important player in both inflammatory and neuro-inflammatory processes, which has gained considerable attention in the past decade and that may represent a key factor in peripheral-central neuro-immune crosstalk. Due to space limitations, we will not review the fundamental importance of alpha-synuclein in immune and neuro-immune processes and crosstalk but will sometimes introduce it where appropriate. The subject is complex and deserves a space on its own to be properly addressed and has been reviewed elsewhere (7–10). Finally, we will consider the importance of LRRK2, a major genetic risk factor for developing PD that is expressed by both neurons and immune cells. For each point we will present and compare data obtained from human and animal studies and underline how they converge to help us improve our understanding of immune crosstalk.

## Neuroinflammation and Microglia in PD

The CNS, long considered an immune-privileged organ, has developed a tightly regulated immune reactivity (11). We now know that insults, such as endogenous danger signals or pathogens, can trigger an immune response in the CNS. Neuroinflammation is the combined response of immune cells present in the brain, including microglia, astrocytes, infiltrating lymphocytes as well as inflammatory factors. Microglial cells and microgliosis seem to play a particular important function in the initiation of neuroinflammation (12, 13). If not controlled or terminated immune reactions may alter brain homeostasis and cause cellular cell death and chronic inflammation (14). Degenerating neurons may themselves release molecules that will spark inflammation (15), triggering a deleterious feedforward loop.

McGeer and collaborators first evidenced the presence of microglial activation in postmortem brains of PD patients suggesting that neuroinflammation may promote neurodegeneration in PD (16, 17). Population-based prospective data has also indicated that the chronic low-dose consumption of non-steroidal anti-inflammatory drugs (NSAID) reduced the risk of developing PD although the protective effect depended on the type of NSAID molecule (18–22). Anti-inflammatory drugs have, however, not yet proven efficacious as anti-symptomatic or disease-modifying treatments (23). Over the past decades neuroinflammatory processes have undeniably been linked to PD but whether they may be a cause, or a consequence of neuronal degeneration remains unanswered (13). Intrinsic damage in degenerating neurons also referred to as cell-autonomous pathological mechanisms may drive their death and was long considered the sole causes of neurodegeneration. Neuronal degeneration may also be a secondary event induced by pathological interactions or signals from neighboring glial cells or immune cell infiltrating from peripheral compartments. The discovery of Lewy bodies containing alpha-synuclein aggregates (24) and the subsequent development of transgenic mouse models expressing alpha-synuclein in astrocytes and displaying PD-like phenotypic dysfunctions (25) supported the existence of non-cell autonomous mechanisms in the disease.

Microglia cells do not originate from blood-derived cells but are established during early prenatal period from yolk-sac-derived progenitors. They remain segregated in and are shaped by the CNS, maintaining self-renewal abilities throughout life (26, 27) without the contribution of peripheral myeloid cells (28, 29). Under physiological conditions microglia constantly surveil the brain parenchyma and provide trophic support to neurons (30–32). Physiological brain homeostasis, including intact barriers that separate CNS from peripheral compartments, regulated expression of soluble factors (TGF-β, Il-4, Il-13, BDNF, NGF) and receptor-mediated cell-cell interactions, all intervene in maintaining microglia under a surveillance-competent phenotype (33). Microglia cells are equipped with receptors to sense endogenous as well as pathogen danger signals (34). Immune mechanisms combine to confer a tight regulation of microglia function in the brain parenchyma. These include, separation from the blood by barriers, soluble factors such as TGF-β, specific interleukins, BDNF etc., direct contact with neighboring cells through receptors, such as the fraktaline receptor CX3CR1, CD200R, MHC II, as well as transcription factors that may regulate activation phenotypes (33). Under pathological conditions, microglia undergo morphological and functional changes and become “activated” (35); they acquire phagocytic phenotype, increase the expression of chemokine and cytokine receptors and are themselves a constant supplier of inflammatory factors (32). Recent evidence clearly indicates that microglia can assume a large variety of phenotypic changes upon activation and show significant regional variability in terms of gene expression profile and functionality that goes well-beyond the simple definition of M1/M2 classification (36).

### PD Patients

**Figure 1:**
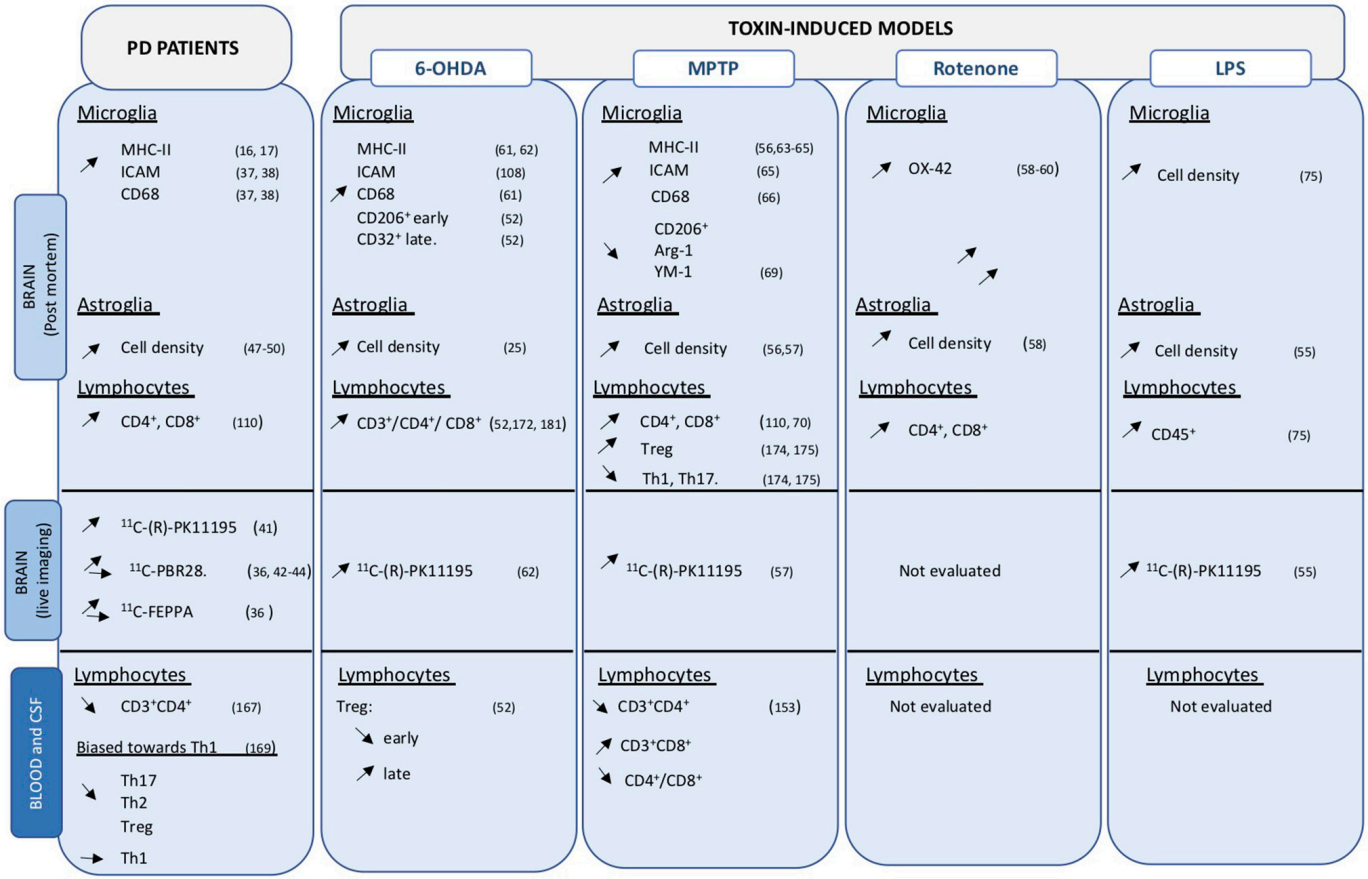
Overview of cellular changes in central and peripheral compartments. Data obtained on PD brain and blood or CSF samples, as well as those from toxin-induced animal models are indicated. Up- and down regulation are indicated by the corresponding arrows.

**Figure 2:**
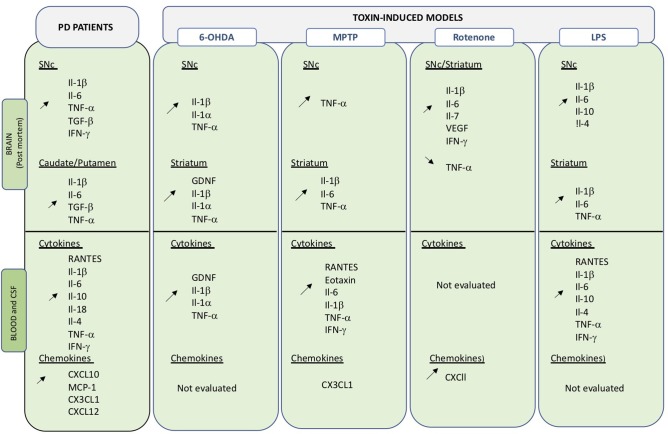
Overview of changes in inflammatory molecules in central and peripheral compartments. Data obtained on PD brain and blood or CSF samples, as well as those from toxin-induced animal models are indicated. Up- and down regulation are indicated by the corresponding arrows.

Activated microglia express major histocompatibility class II (MHC-II) markers that present peptides to effector cells including T lymphocytes. In humans these include human leukocyte antigen (HLA)-DR, HLA-DQ, and HLA-DP molecules. Presentation is key to the engagement of adaptive immune, which in turn can further sustain inflammatory processes. In postmortem PD brains, HLA-DR^+^ microglia were observed in the SNc and striatum (16, 17) (Figure 1), mainly associated with neurons containing LB and damaged neurons (37). They also expressed intracellular adhesion molecule (ICAM), the scavenger receptor TLR2 and the lysosomal marker CD68 (37, 38). Interestingly, pro-inflammatory factors, including IL-6 and TNF-a, were expressed in MHCII+ cells (37) (Figure 2).

Single nucleotide polymorphism in the MHC-II locus has been associated with increased risk of developing PD (39) and genome-wide association studies (GWA) (39) have noticeably implicated HLA cell-surface complexes as fundamental to trigger the adaptive immune system in the frame of PD pathology (40).

Positron emission tomography (PET) using ^11^C-(R)-PK11195), a radioligand that binds to the 18-kDa translocator protein (TSPO) mainly expressed on “activated” microglia (41), evidenced increased binding in various brain regions in PD patients compared to healthy controls (36, 42–44). Inconsistent data was obtained with a panel of radioligands, possibly as a consequent of polymorphism-linked difference in TSPO binding affinity of the ligands (45, 46). Thus, while PET analysis can be used to confirm microgliosis in PD, ligands do not have the ability to distinguish among the phenotypic diversity of activation states and do not allow correlation between imaging and disease severity or progression.

Astrocytes are the most abundant cells in the brain. They regulate many brain processes including glucose metabolism (47). They play a fundamental role in maintaining brain homeostasis and providing energy and support to neurons (48). Their role in PD pathology still not well-understood but an elevated cell density and phenotypic changes are observed in astrocytes in postmortem PD brains (49, 50). Astrocytes also contribute to the blood-brain-barrier that is disrupted in patients with PD (51).

### PD Animal Models

Glial activation, both astrocytes and microglia, has been consistently observed in toxic animal models of PD, including the 6-OHDA rats and mice (52–54), LPS (55), MPTP treated animals (56, 57), and rotenone models (58–60) (Figure 1).

Intracerebral injection of the neurotoxin 6-hydroxydopamine (6-OHDA) in rodents (rats and mice) causes nigrostriatal neurodegeneration and induces a strong glial activation (microglia and astrocytes) in the striatum and substantia nigra (SN). Profile, localization and time of neuroinflammation depend on the site of injection. Increased MHC-II and CD68 expression is detected rapidly after 6-OHDA injection in the medial forebrain bundle, in particular in microglia located nearby neuronal cell loss (61, 62). In 6-OHDA models, microgliosis seems to be a transient phenomenon that peaks shortly before neurodegeneration and then slowly reverts to an apparently normal phenotype. Intrastriatal injection of 6-OHDA in mice causes a rapid increase of TNF-α in microglia indicating an inflammatory-prone phenotype (54).

The MPTP neurotoxin induces persistent microgliosis in non-human primates (NHP) evidenced by enhanced HLA-DR^+^ microglia (56) reminiscent of phenotypes observed in PD brains (16, 17). In MPTP-treated NHP chronic microglial activation is still present years after MPTP intoxication (56, 63).

A more transient activation of microglia cells is observed in rodents depending on the MPTP regimen and paradigm (56). In a chronic rodent model of intoxication microglia activation is detected well-before neurodegeneration when non-motor dysfunctions, including hyposmia are already present and persists for at least 6 months (64). Upregulation of MHC-I, MHC-II, and ICAM-1 are detected transiently in MPTP mice (65). Increased CD68 expression in microglia located close to neuronal cell death is also observed in MPTP mice (66). Interestingly, the neuro-toxin induces a down regulation of anti-inflammatory markers, including CD206, Arg-1, and YM-1, in the SN suggesting a shift toward a more inflammatory-prone phenotype of microglial cells (67).

Rotenone, a naturally occurring substance largely used in organic agriculture until its prohibition in 2008, has been linked to the development of PD (60) and has been used to develop rodent models of the disease through different administration paradigm including intracerebral or intraperitoneal injection and intragastric administration (68, 69). Consistent neuroinflammation and microglial activation is observed in rotenone models (58–60, 70).

Peripheral or intracerebral injection of the bacterial endotoxin lipopolysaccharide (LPS) causes microgliosis that precedes neuronal cell death (71). LPS acts through interaction with the Toll-like receptor 4 (TLR-4) and is a potent inducer of peripheral (72) and central immune cells (73, 74) but has no direct effect on neuron. LPS injection has been used in numerous toxic-induced or transgenic models of PD and accentuates neurodegeneration and neuroinflammation (48, 49). Recent data indicated that peripheral injection of LPS induces microglial activation that precedes and peaked just before neurodegeneration and then slowly decreased. Interestingly, at later time points a shift toward a more anti-inflammatory profile was observed in microglia (Arg-1^+^ cells) corresponded to cessation of neurodegenerative processes (75).

Numerous transgenic models of PD have been developed in particular model overexpressing wild type or mutant forms of the protein alpha-synuclein using different promoters (25). The thy-1 a-syn transgenic model, the best characterized syn model (61), shows early and progressive increase of activated microglia specifically detected in the SN and striatum and that precedes nigrostriatal neurodegeneration (76, 77). Similarly, viral vector-driven overexpression of a-syn models (AAV a-syn models) have been developed in rodents (78) and primates (79). Robust microglial activation is also observed in AAV a-syn models in rodents (80–82) and in primates (83) and are consistent with neuroinflammatory features observed in PD patients including MHC-II upregulation. A paper by Ferreira and Romero-Ramos (84) extensively reviews the role and crosstalk between a-syn and microglia in PD and because a space limitation this topic will not be further addressed here.

Evidence and data obtained in PD patients and animal models of PD (Figure 1) clearly converge and sustain the importance of neuroinflammation in the disease.

## Communication Routes Between the Periphery and the Brain

Crosstalk between systems implies and requires the existence of communication routes. Neurons in the brain are protected from adverse effects of peripherally borne insults by several barriers, including the blood-brain barrier (BBB), the blood CSF barrier (BCSFB) and the meninges. They have different permeability to substance and cells. Under physiological conditions only few leukocytes are observed in the CNS. These barriers represent physiological and selective entrance to the CNS, yet at the same time they are also a niche where blood-derived immune cell may distantly modulate or affect brain homeostasis (85).

The BBB is formed of endothelial cell tight junctions and a layer astrocytes end-foot. BBB alterations may be linked to aging, by far the most relevant risk factor for developing PD. Astrocytes are important players in maintaining an intact BBB and age-related changes in astrocytes may modify BBB permeability (86, 87). Astrocytes also release numerous soluble factors, including monocyte chemoattractant protein-1 (MCP-1), which favors the recruitment and infiltration of monocytes from the periphery into the brain. The CSF, filling the space in between, contains self-maintained resident myeloid cells of embryonic origin (88). Under physiological conditions, the BBB allows the passive diffusion of water and lipophilic molecules and the selective transport of molecules necessary for neural function, such as glucose and amino acids, but does not permit cellular infiltration to the brain. Dysfunction or disruption of tight junctions can cause leaky BBB and exposes the brain to blood-borne substances.

The BCSF is formed by the choroid plexus (CP) that produces and distributes the CSF throughout the CNS (89). It is formed of tight junctions and epithelial cells, which express trafficking molecules (85, 90, 91) These may sense and response to signals secreted by immune cells present both in the CNS and the stroma. The presence of epithelial vs. endothelial cells renders the BCSF less impermeable than the BBB and trafficking of a low number of T cells, in particular CD4^+^ memory T cells, is possible even under physiological conditions (92).

The meninges surround the brain and the spinal cord and contain the myeloid- and T-cell-populated CSF in the subarachnoid space (88, 93). Similarly to the BCSF, they have a different anatomical structure than the BBB that could allow migration of immune cells into the brain parenchyma (94).

Recent evidence indicates that a selected population of resident cells, including T cell subsets, NK cells, B cells and dendritic cells, are present in those brain boundaries, the “brain interface,” mostly located in the meninges and the BCSF. These cells may serve as communication bridges to and from the brain (95) and have been defined in depth recently (96). Expression of CD44 is crucial for cell motility and is not present in brain resident myeloid cells (96).

Pathological events, in the CNS or in peripheral systems, may modulate barrier integrity leading to important functional dysregulation and opening of tightly regulated communication routes causing unwanted crosstalk and passage of noxious molecules or cells that may sensitize or worsen existing conditions. Leakage could potentiate existing neuroinflammatory processes by allowing infiltration of peripheral cells into the brain parenchyma. It could also be caused by neuroinflammation itself.

### PD Patients

BBB disruption has been reported in PD patients (97, 98). Post-mortem brain samples show accumulation of blood-derived proteins (fibrinogen, IgG) in the striatum and globus pallidus (51, 99). Microvascular degeneration, disrupted and damaged tight junctions, changes in the capillary basement membrane of the subthalamic nucleus, as well as red blood cell extravasation in striatum have been documented. Aberrant angiogenesis in the SN, locus coeruleus and putamen also support alterations in BBB (100, 101). Increased of blood-derived albumin in CSF and of IgG CSF: serum ratio (102, 103) are also indicative of barrier dysfunction. Live neuroimaging studies have evidenced disruption of BBB integrity in basal ganglia (104) and deep cortical gray matter regions and white matter (105), as well as diminished P-glycoprotein function (97).

### PD Animal Models

Existing data in animal models confirm the presence of barrier alteration although this pathological data has not been systematically evaluated in all models. Injection of LPS causes BBB disruption and loss of TH-positive cells in rodents (23, 106). When present, BBB disruption occurs mostly at the site of dopaminergic cell loss but the causes and sequence of events leading to BBB permeability changes are still a matter of debate.

Altered expression of cerebral adhesion molecules, possibly reflecting BBB alterations have been detected in 6-OHDA rats (105, 107) and have been associated with concomitant alterations of peripheral molecules (108). Increased BBB permeability has also been observed in the striatum and SNC following intrastriatal injection of 6-OHDA (109). It has also been suggested that brain accumulation of iron measured in 6-OHDA animals is partly due to altered BBB (107).

Upregulation of adhesion molecules in important for infiltration of cells has been observed in MPTP models (rodents and NHP) (110, 111). Peripheral inflammation itself may cause BBB alterations, thus favoring or sustaining neurodegeneration. Inflammatory mediators, produced systemically, or within the brain, can signal through the endothelial cells causing alteration in tight junctions' structure thus modifying BBB permeability. Increased number of blood vessels and endothelial cells have been described in proximity of degenerating neurons in MPTP-treated NHP (112). Changes in phospho-glycoprotein functionality suggestive of BBB alterations have also been observed in MPTP-treated NHP (113). Therapeutic strategies that prevent BBB leakage through activation of CB2 receptors have been shown to reduce dopamine neuron loss in the MPTP model (114). However, it remains unclear whether increased BBB integrity is a causal effect or the result of reduced neuronal cell loss.

No clear evidence of BBB alteration, measured by fluorescein leakage to the brain, has been detected in the rotenone model (115).

Globally, evidence obtained from human and animal studies clearly points to a dysregulation of barriers between the peripheral and central compartment. It remains to be determined if increased BBB integrity is a causal effect or the result of neuronal cell loss and neuroinflammation.

## Inflammatory Markers

Peripheral inflammatory markers including cytokines and chemokines are critical signaling molecules in the modulation of the immune system and can affect both peripheral and central systems. Cytokines and chemokines are actively transported across the BBB by saturable transport and any variations in expression levels in the blood may directly or indirectly impact on CNS function (116). Peripheral inflammation could thus be an important contributor in the etiology of PD as well as in disease progress (Figure 2).

### PD Patients

Increased levels of inflammatory markers were already detected in PD post-mortem samples in the late 1990's (Figure 2) (117–121). Changes in cytokine/chemokine levels have then been largely evaluated in PD patients biofluids (blood, serum plasma, CSF) and measurements show divergences of pro-inflammatory and anti-inflammatory profiles compared to healthy subjects (122–126). Qin and collaborators have recently performed a systemic review and meta-analysis of published data to investigate alterations of peripheral cytokines in PD patients (127). Findings from 25 peer-reviewed publications, including 1,547 PD patients, and 1,107 healthy controls, confirmed that patients present an increased inflammatory-prone response and allowed the identification of elevated levels of pro-inflammatory factors, including RANTES Il-1β, Il-2, Il-6, TNF-α, and C-reactive protein. Interestingly, Il-1β is an important central downstream effector of inflammasome activation, an inflammatory mediator that is attracting considerable attention in neurodegenerative diseases (see below section Inflammasome). Altered levels of IFN-γ and Il-8 were also detected, although in a limited number of small studies (122, 128). Similarly, alterations in levels of the anti-inflammatory cytokines Il-4 and Il-10 were detected (118, 122). Il-10 is thought to oppose action of proinflammatory cytokines but may also be involved in B cell survival and activation as well as IFN-γ production. Recently, some of the above-mentioned cytokines have been correlated with specific PD phenotypes (129). High Il-10 was related to non-tremor and late onset PD, while Il-6 correlated with longer disease duration and TNF-α with disease progression. Correlation between RANTEs and disease severity (130) as well as TNF-α blood levels and non-motor symptoms have also been suggested.

Chemokines have been less commonly assessed in blood or CSF of PD patients, but recent data suggest that elevated CXCL10 levels may be related to worsening of cognitive functions in PD patients (131). Similarly, changes in MCP1 (CCL2), CXCL10 and CX3CL1 have been detected (132, 133). Changes are also observed in the CSF, in particular increased levels of Il-2, Il-6, TNF-α and MCP-1 have been measures (134). Importantly, increased levels of Il-1β, Il-6, and TNF-α, have also been detected in PD brains (135, 136), suggesting that changes in both systems may be correlated.

### PD Animal Models

Few studies have addressed the modulation of inflammatory factors in peripheral compartments, including blood and CSF, in animal models of PD. Nonetheless, results obtained in animal models also recapitulate the convergence of peripheral and central changes in inflammatory molecules.

In 6-OHDA rodents blockage of TNF-α or Il-4 can reduce neurodegeneration (23, 137), while it is exacerbated by treatment with systemic Il-1β (138, 139). Interestingly, a similar worsening effect was observed in DJ-1 KO (140) and Parkin KO mice (141) for which neurodegeneration of dopaminergic neurons is usually absent (25). A delayed increase of striatal Il-1β mRNA levels is observed following 6-OHDA administration (142). Modulation of Il-1α, Il-1β, Il-6, and GDNF levels is observed at different time points both in serum CSF and brain extract of 6-OHDA rats (53, 143) It is possible that the time course analyses (24 h, 7 days, 4 and 8 weeks after toxic insult) in the various studies does not allow the detection of transient up- or down-regulation of the inflammatory markers.

CSF and serum concentration of TNF-α and IFN-γ are elevated in MPTP-treated NHP, even several years after intoxication (144, 145). Confirming the importance of the two cytokines, KO mice lacking IFN-γ or TNF-α receptors are protected from MPTP neurotoxicity (144, 146–148). Elevated levels of Il-1β TNF-a and Il-6 are also consistently observed in serum and brain following MPTP administration in mice (67, 149–153).

More recent evidence from the acute MPTP mouse model, indicates a transient increased in both brain and serum levels of RANTES and eotaxin (154, 155). RANTES is known to induce migration and homing of lymphoid cells (156) and as indicated above is also increased in serum of PD patients (127). Eotaxin is an important factor involved in infiltration of mononuclear cells at inflammation sites (157). Biweekly injection of RANTES and eotaxin induced a continuous activation of neuroinflammation and T-cell infiltration as well as persistent neurodegeneration, while functional blocking antibodies to the two factors reduced T-cell infiltration, neuroinflammation and neurodegeneration. Interestingly, increased RANTES and eotaxin levels, as well as T cell infiltration have also been detected in the serum of MPTP-treated NHP (158). Blocking RANTES and eotaxin expression could significantly reduce neuroinflammation. Unfortunately, effect on neurodegeneration of RANTES blockade was not assessed in this study.

Animals that received repeated i.p. injection of LPS presented with a significant loss of dopaminergic neurons that peaked at 19 days and remained stable thereafter. An immediate increase in pro-inflammatory cytokines, Il-1β, Il-6, and TNF-α was detected in the brain. Interestingly, this pro-inflammatory upregulation preceded neurodegeneration, was then reduced when maximal neuronal cell loss was detected, and switch to a more anti-inflammatory profile (Il-10) as cell loss ceased and stabilized (75). This sequential modulation from a pro- to an anti-inflammatory phenotype may represent an interesting target to arrest and resolve chronic neuro-inflammation.

## Lymphocyte Infiltration

In a normal adult brain, the crosstalk between the peripheral immune system and the brain is transient, and there is no evidence that it may lead to the central neuroinflammation. As mentioned above under physiological conditions, only few leukocytes are observed in the CNS. A growing body of evidence suggests that in chronic neurodegeneration, not only are the brain-resident microglia activated (159, 160), but they may be “primed” by previous or ongoing systemic inflammation, leading to the exaggerated synthesis of pro-inflammatory molecules (161–163). Numerous studies showed that microglial cells can be activated by the chronic infiltration of peripheral inflammatory T cells (164, 165), as well as various toxic molecules circulating from the peripheral tissue to the brain (166). T lymphocytes are an essential component of adaptive immunity and collaborate with B cells to produce an immune response.

### PD Patients

T cell populations are altered in peripheral compartments and invade the CNS in PD and may contribute to neuronal degeneration and disease progression. Infiltration of CD4^+^ and CD8^+^ cells have been observed in post-mortem analyses of PD brains (110) (Figure 1).

In peripheral blood of PD patients, alterations of the adaptive system are detected showing a decrease in both B and T lymphocytes, together with alterations in components of innate immunity including increased natural killer cells and neutrophils levels. In particular, a reduction in CD3^+^/CD4^+^ lymphocytes is consistently described, while the number of CD8^+^ cells remains largely unchanged in blood of PD patients (167).

CD4^+^ T cells can acquire different phenotypes corresponding to different inflammatory states. Pro-inflammatory T helper (Th) cells include Th1 that produce IFN-γ and TNF-α, and Th17 cells, producing Il-17 and Il-22. Anti-inflammatory cells include Th2 that release Il-4, Il-5, and IL-13, and regulatory T cells (Treg) that are fundamental modulator of T cell activation. Kustrimovic and collaborators have recently indicated that the balance among different T-cell phenotypes in the blood of PD patients is biased toward a more Th1-response, with a reduction in the number of Th17, Th2, and Treg, but not Th1 cells (168). This imbalance was further reflected by a Th-1-prone polarization in response to specific inflammatory stimuli, observed *in vitro* in lymphocytes from PD patients but not healthy volunteers. The reduced efficacy of PD Treg cells in controlling the release of pro-inflammatory cytokines by effector T cells (169) is a likely contributing factor that further amplifies this Th1-prone profile of peripheral T cells in PD.

### PD Animal Models

Evidence in toxin-induced animal models corroborates data obtained in PD patients and sustains the important function of T cell subsets in neurodegenerative processes in PD (Figure 1). Infiltration of T cells, in particular CD4^+^ and CD8^+^ infiltration in the brain parenchyma, has been documented in numerous animal models of PD, including MPTP mice (110, 170), intragastric rotenone PD model (171), as well as in 6-OHDA PD models (52, 172). Much information on T-cell infiltration has been obtained using the MPTP mouse model combined to a variety of transgenic models. Rag1^−/−^ mice, which lack mature lymphocytes, and Tcrb^−/−^ mice, which lack T cell receptor β, are more resistant to acute MPTP toxicity compared to control mice (173, 174). Similarly, administration of MPTP to CD4^−/−^ mice induced less prominent dopaminergic cell loss compared to that observed in CD8^−/−^ animals (110). Altogether, these data indicate the importance of T lymphocyte infiltration and sustain a prevalent function of CD4^+^ over CD8^+^ lymphocytes in the MPTP-induced neurodegeneration processes.

The Th1-prone imbalance together with the reduced Treg efficacy observed in PD patient blood, combined with the importance of anti-inflammatory action and regulation of Treg in neurodegeneration is further sustained by experiments involving adoptive transfer of T cell subsets in MPTP mice. Transfer of Treg cells reduced neuronal cell loss, while transfer of Th1 or Th17 increased neurodegeneration (174, 175). In the same line, immunization with bacillus Calmette-Guerrin that favors Treg activation had a protective potential in MPTP mice insult (176). Chung and collaborators also reported that neuroprotective potential of bee venom immunization in MPTP mice could be linked to a global reduction of CD4^+^ infiltration accompanied by a relative increased proportion of Treg cells in the brain parenchyma (177). Reduction in the number of lymphocytes in MPTP mice has been reported as early as 1992 (178) and confirmed by recent data reporting a global reduction in the number of CD3^+^ with reduced CD3^+^CD4^+^ but increased CD3^+^CD8^+^ cells (153).

Infiltration of T-lymphocytes has also been observed in 6-OHDA mice and rats PD models together with time-dependent neuroinflammation (52, 179). Blood of 6-OHDA animals showed an initial decrease in Treg cells that progressively returned to normal values. Interestingly, reduced Treg levels at the peripheral level corresponded to a phenotypic shift in microglial activation, from an anti-inflammatory phenotype (CD206^+^) to a more pro-inflammatory (CD32^+^) phenotype, as well as with the reduction of neuronal cell loss in the SNc, further suggesting an important modulatory role of Treg cells in the neuronal cell loss and neuroinflammatory (53).

Considering the close interrelationship between T cells and microglia cells (180), therapies that change T cells may directly modulate microglial phenotype and vice and versa. For example, stimulation of the regulatory function of CD4^+^ cells infiltrating the brain may represent and therapeutic strategy to limit neurodegeneration.

## Monocyte/Macrophages

As described above the presence of infiltrating lymphocytes in the CNS is well-documented both in animal models of PD and in post-mortem analyses of PD brains. Differently, a role for monocytes/macrophages in PD remains unclear but evidence suggests that they may also be contributing actors to the disease. Macrophages and monocytes are important players in the regulation of immune reaction in peripheral compartments and can pass the BBB to enter the brain where they may participate in regulation of central neuroinflammatory process (181).

Monocytes are short-lived myeloid-derived cells that continuously generated from bone marrow precursors (182). Monocytes circulate in the blood and tissues and do not proliferate under physiological conditions. They are key components of the innate immune system, express cell surface receptors as well as pathogen recognition receptors, and can produce cytokines. During inflammation they may migrate to inflamed tissues and differentiate into dendritic cells or macrophages (27, 183). Under physiological conditions, monocytes are constantly renewed from the myeloid repertoire while microglia renew themselves without the contribution of peripheral myeloid cells (28, 35).

Circulating monocytes can be found in the brain parenchyma only following BBB disruption caused for example by irradiation and bone marrow transplantation (184). Parabiosis experiments (26, 185) indicate that there is no infiltration of monocytes in the brain under physiological conditions. In neurodegenerative diseases, monocytes may infiltrate the brain and join microglia. As microglia they also undergo phenotypic changes, rapidly acquire a macrophage-like phenotype but never fully gain a microglial identity (186). Until recently, it was difficult to distinguish infiltration monocytes from endogenous microglia. Recent data indicates that the chemokine receptor CCR2, required for cellular infiltration, is expressed on blood monocytes but not on resting or activated microglia and that the reverse is true for the fraktaline receptor CXC3CR1 (187, 188). This differential expression of cell surface markers favors the distinction between monocytes and microglia population in the brain parenchyma during a short time-window before infiltrating monocytes turn into tissue macrophages and downregulate CCR2 (189).

A specific population of macrophages are located at the brain interface, including meninges and CP; they have the same ontogenetic origin as microglia and differentiate through fine-tuned processes to give rise to separate cellular population with distinct profiles (190). Brain interface macrophages can be replenished by circulating cells but mostly originate from embryonic yolk sac and are maintained by self-renewal (88). They express microglia markers including CD11b, CX3CR1 and are different than circulating monocytes. The role of these perivascular macrophages is still under study.

### PD Patients

Recent data indicates that, in early stage PD patients, disease-specific gene expression in peripheral monocytes may correlate with disease severity (191). Interestingly, genes relating to leukocyte migration and regulation of immune responses were found to be enriched in PD monocytes. Of particular interest, LRRK2 expression was highly upregulated in monocytes (see the section LRRK2: a genetic factor and an immune mediator in PD). Different gene expression patterns in monocytes are observed when looking at distinct disease stages (192) suggesting that monocytes may represent an important population to identify disease progression markers in PD.

### PD Animal Models

MPTP treatment in mice increases the number of circulating monocytes (193). Infiltration of CCR2^+^ monocytes has been detected in the brain parenchyma of CCR2-GFP reporter mice following acute MPTP treatment (194). Interestingly, monocyte infiltration was transient and occurred before infiltration of T cells. In this acute model, blocking CRR2^+^ had no effect on MPTP-induced neurodegeneration. Precise contribution of monocytes infiltration to neuronal cell death still needs to be clearly demonstrated and more chronic states of infiltration may be needed.

## Inflammasomes and Parkinson's Disease

Innate immunity is the first line of defense of the organism. It has evolved to recognize conserved pathogen molecular sequence (pathogen-associated molecular pattern—PAMP) through a set of receptors, the pattern recognition receptors (PRR). PRR can also be activated by damage-associated molecular patterns (DAMP). Recognition of PAMP or DAMP by PRR normally triggers transcriptional activation and neo-synthesis of proteins. Inflammasomes react to specific PRR signal, trigger caspase 1 activation, which in turn causes the maturation and release of the pro-inflammatory cytokines Il-1β and Il-18.

The inflammasome is a macromolecular complex formed through the oligomerization of a receptor, an adaptor, and caspase-1, the effector of the complex. Inflammasome receptors belong to several families, including the nucleotide-binding domain and leucine-rich repeats containing receptor family (NLR). In the past years NLRs have emerged as key sensors and regulators responding to PAMP and in particular DAMP produced under non-microbial inflammatory conditions (195). NLRP3 is the most characterized and studied inflammasome receptor and NLRP3 alterations have been linked to several pathologies, including neurodegenerative diseases (196). Evidence indicates that inflammasomes are important players in both peripheral and central innate immunity (197) and need to be tightly controlled to avoid overt inflammatory activation (198, 199).

### PD Patients

**Figure 3:**
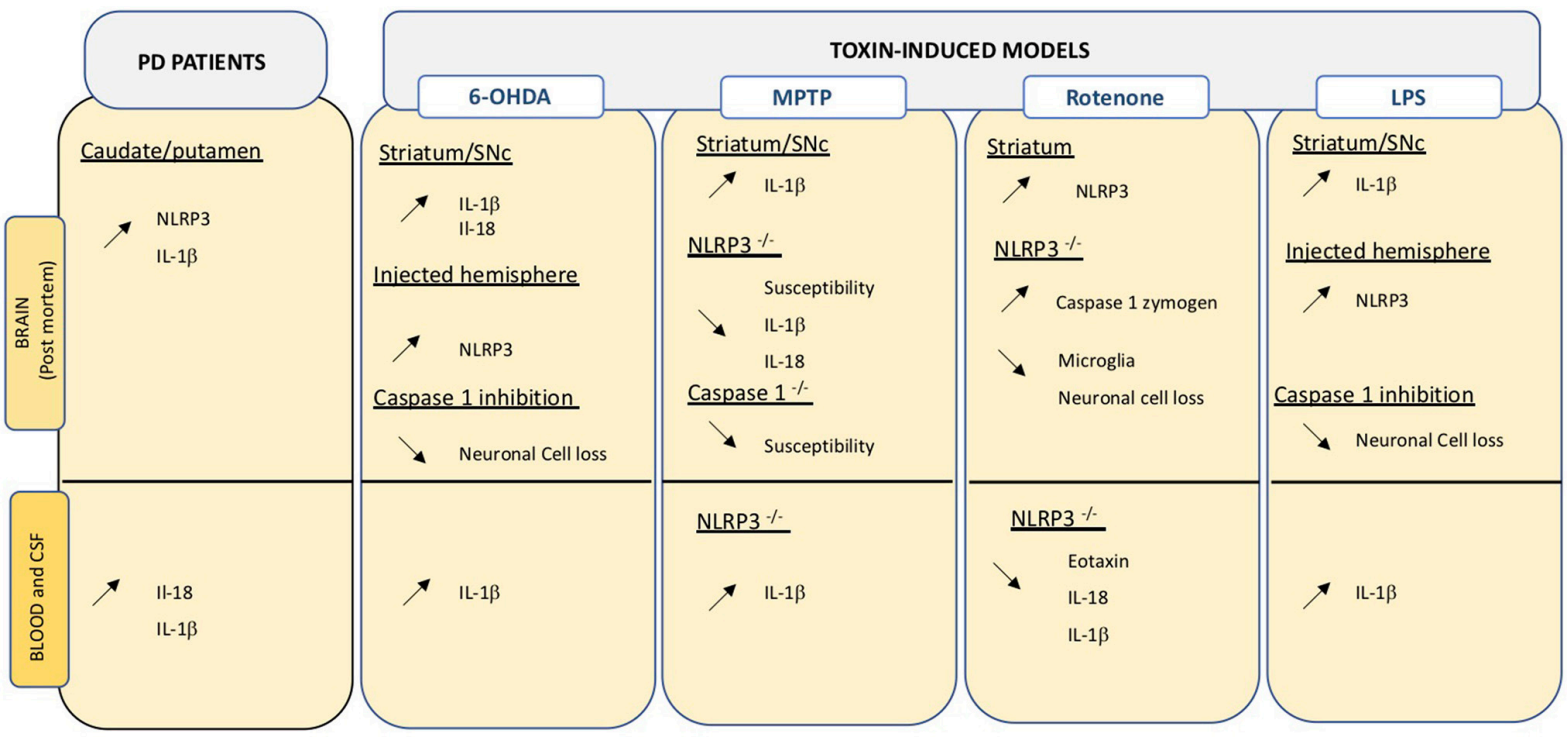
Overview of changes relating the inflammasome in central and peripheral compartments. Data obtained on PD brain and blood or CSF samples, as well as those from toxin-induced animal models are indicated. Up- and down regulation are indicated by the corresponding arrows.

Post-mortem analysis of confirmed idiopathic PD brains revealed high NLRP3 protein expression in surviving neurons (200). Downstream effectors of NLRP3, namely IL-18 and IL-1β are increased in CSF and serum of PD patients (201). IL-1β is a key cytokine in PD and increased levels are detected in central and peripheral compartments both in PD patients and in animal models of the disease (Figure 2). Considering the close link between NLRP3 and IL-1β maturation, inflammasomes may be an important linking bridge between peripheral inflammation and central PD pathology (Figure 3).

A SNPs variant (rs7525979) of NLRP3, linked to altered stability of inflammasome assembly, is associated with significantly lower risk of developing PD (200). Interestingly, NLRP3 polymorphism has recently been associated with inflammatory bowel disease [IBD (202)]; IBD being a recently identified risk factor for PD development (203). Sustaining this link, recent retrospective analyses indicate that anti-TNF-α therapy to treat IBD reduces the incidence of PD, with a 78% reduction of risk in treated compared to non-treated patients (204). Thus, peripheral and central inflammation may be linked through activation of inflammasomes. Thus, a specific genetic susceptibility bridges PD and inflammation in the gut. The importance and involvement of the gut-brain axis PD pathogenesis has drawn considerable attention in the past decades. The Braak theory suggests that PD pathology may initially be triggered in the gastrointestinal (GI) tract and then spread to the brain via the vagus nerve. While the Braak theory is still a matter of debate (205, 206), the dysregulation of the gut-brain axis is well-established (207). A majority of patients suffer GI disorders that often appear before the onset of any motor symptoms (208). Today, there is no consensus on whether GI disturbances occur as a consequence of neurodegeneration in the CNS, the enteric nervous system, or both, or because of a yet unknown pathological process. Evidence supporting a contribution of chronic intestinal inflammation in PD has been covered elsewhere (207, 209, 210) and will not be reviewed here.

Several pathologic features characteristic of PD are linked to inflammasome regulation and activation further sustaining its importance. Indeed, dopamine (DA), the key neurotransmitter in PD, acts on almost all peripheral blood cells, which knowingly express dopamine receptors (211). DA has been shown to be an endogenous modulator of inflammasome by promoting NLRP3 ubiquitination and degradation (212). DA can inhibit LPS-induced NLRP3 activation in mice (212). Inflammasome can also be triggered by α-syn (213). Mitochondrial alteration is a common defect normally observed in most cell compartments in PD patients (214). Mitochondrial stress induced by rotenone can prime the activation of NLRP3 pointing out a link between mitochondrial defect and inflammasome activation (215, 216).

### PD Animal Models

Evidence in an animal model of PD indicates that IL-1β is primarily induced by NLRP3 activation in brain and microglia (217). Multiple studies in animals have shown that blocking NLPR3 can block development of PD phenotypes. Inhibition of inflammasome in MPTP-treated mice can reduce neuronal cell death (218). NLRP3^−/−^ mice are less susceptible to MPTP compared to wild type animals and show reduced Il-1β and Il-18 production in serum (212). On the same line, mice lacking Caspase-1, the inflammasome effector, are less susceptible to MPTP (219). Inhibition of caspase-1 also reduces susceptibility to intracerebral administration of LPS or 6-OHDA in rats (220). Intragastric and ip exposure to rotenone increases striatal NLRP3 levels in treated mice (171, 221) and leads to caspase 1 cleavage, as well as neuroinflammation. Interestingly, the same treatment in mice lacking NLRP3 does not induce an inflammatory response and reduced neuronal cell death, typically induced by the neurotoxin, is detected (171) (Figure 3).

A large number of cytokines can be produced by the liver, which seems to be an active indirect participant in inflammatory processes. Aside from increased NLRP3 activation in the brain, MPTP-treated mice also present NLRP3 changes in liver and bone marrow-derived macrophages (BMDM) (222). In these mice, the selective liver-directed downregulation of NLRP3 reduced hepatic NLRP3 levels, as well as levels of proinflammatory cytokines in serum and brain. Specifically, Il-1β and Il-18, but not the anti-inflammatory Il-4 and Il-10 cytokines, were impacted, suggesting the induction of an anti-inflammatory prone environment. Decreased hepatic inflammasome activation was accompanied by reduced neurodegenerative and neuroinflammatory processes. Interestingly, hepatic alterations have been previously described in 6-OHDA treated animals (143) and it was suggested that brain-liver axis may intervene in a feedforward loop in which neurodegeneration caused hepatic alteration that in turn exacerbated neurodegeneration. Inflammasome components have not been evaluated in this study but could represent an important mediator in peripheral-to-central transmission of inflammation and vice and versa. Interestingly, BMDM isolated from animals receiving hepatic inhibitory vectors displayed reduced release of Il-1β and caspase 1 in the absence of changes in NLRP3 expression suggesting an indirect effect produce by hepatic inhibition.

The study of NLRP3 is important because inflammasomes are the core of sterile inflammation associated with exposure to chemicals, proteinopathies as well as stress that are all part of the multifactorial panel influencing incidence of PD. A more thorough understanding of inflammasomes and effects of inhibition of NLRP3 in existing models together with evaluation of peripheral levels of factors is warranted across models and in peripheral blood of PD patients.

## LRRK2: a Genetic Factor and an Immune Mediator in PD

Leucine—rich repeat kinase 2 (LRRK2) is a large 286 kDa protein that contains several distinct functional and protein-protein interaction domains. This inherent structure suggests that LRRK2 interacts with different partners in different cells and may modulate numerous cellular functions (223) and pathways. LRRK2 mutations are associated with a dominant form of familial PD that is similar in presentation and age of onset to idiopathic PD. Up to 40% of familial PD is linked to LRKK2 mutation (224). Interestingly, the LRRK2 locus is also a major genetic susceptibility factor in idiopathic PD (225). The two most common PD-related LRRK2 mutations, G2019S, and R1441G/C, cluster within the kinase and GTPase domains that are surrounded by large interactions sites (226) but the physiological importance of LRRK2 itself and the contribution of mutations to PD pathology remain unclear. LRRK2 mutations have an incomplete penetrance in PD patients and most rodent transgenic models developed to date (http://www.neurodegenerationresearch.eu/models-for-parkinsons-disease/in~vivo-mammalian-models/lrrk2/) show no or little evidence of any neuronal cell loss, strongly indicating that other factors, environmental or genetic, must intervene to trigger neuronal cell loss.

### PD Patients

Large GWA studies have detected common LRRK2 variants that confer increased (N2081D) or reduced (N551K or R1398H) risk to develop Crohn's disease, a subtype of IBD (227). The N2081D, but not N551K and R1398H variants increased LRRK2 kinase activity. As mentioned above IBD is a recently identified risk factor for PD. Effects of the variants on IBD also correlated with an increased and reduced risk to develop PD. Both diseases share inflammation as a common denominator and may therefore share common disease mechanisms, in which LRRK2 may be an important hub.

LRRK2 is expressed in both neurons and cells of the innate and adaptive systems (228, 229), where it may become upregulated following microbial or viral infection. Expression levels of LRRK2 protein are increased in B and T cells, as well as in monocytes of PD patients compared to control subjects (230). Interestingly, LRKK2 levels were elevated specifically in CD16^+^ pro-inflammatory monocytes, with a slight increase also observed in T effector cells. *In vitro*, stimulation of PD patient-derived monocytes induced stable and long-lasting activation of HLA-DR, while only short-lived HLA-DR activation was observed in control monocytes. HLA-DR is part of the MHC-II locus that is highly polymorphic and is involved in antigen presentation required for CD4^+^ cell activation. Opposite correlations between LRKK2 and HLA-DR expression, positive or negative, were observed in monocytes from PD patients and control subjects, respectively (230). SNPs in the HLA-DR region have been associated with increased risk of developing PD (39, 225, 231, 232). The combination of specific HLA polymorphisms and pesticide exposure seem to favor the induction of a more pro-inflammatory-prone CD4^+^ activation (233). Thus, LRRK2 may be an important factor that intervenes at the immune interface and may favor a pro-inflammatory-prone environment in PD patients as well as in animal models (Figure 2).

### PD Animal Models

LRRK2 deficient rats show significantly increased percentage of CD4^+^ and CD3^+^ cells, but not CD8^+^ in the spleen, as well as a reduced percentage of B cells compared to wild type animals (234). Interestingly, LRRK2 KO rats challenged with α-syn overexpression or intracerebral LPS administration are protected from neurodegeneration normally occuring in wild type animals (235). Transgenic mice overexpressing pathogenic LRRK2 mutations, R1441G or G2019S, do not normally present any evidence of neuronal cell loss or neuroinflammation in the SNc. Recently, Kozina and collaborators showed that systemic LPS-induced inflammation triggered significant loss of TH-positive cells only in mice overexpressing a mutant form of the human LRRK2 protein (236). No neurodegeneration was induced by LPS in mice overexpressing the human wild type LRRK2 protein, strongly suggesting a role of LRRK2 mutations in pathological mechanisms leading to cell loss. Interestingly, no lymphocyte infiltration was detected in transgenic LRKK2 mutant animals. Analysis of peripheral immune reaction indicated that systemic LPS administration in the context of mutant LRRK2 triggered significant increase in peripheral cytokines that exacerbated neuroinflammation in the brain, increased LRRK2 expression in neurons and caused neurodegeneration. Interestingly, time-dependent increase of peripheral expression of numerous immune factors, including IL-1β, IL-6, IL-10, RANTES, CXCL1, were detected in LPS-treated mutant LRRK2 mice factors. These factors are reminiscent of alterations observed in the blood of PD patients (see the section “Inflammatory markers” above).

## Discussion

The etiology of PD pathogenesis is still largely unknown, and evidence strongly indicates that combinations of multiple factors are involved in triggering neurodegeneration. Neuroinflammation and systemic inflammation have been implicated in PD pathogenesis and appear as key aggravating factors. Yet it remains unknown if inflammation and immune dysfunctions mediate PD or if PD mediate immune dysfunction in both peripheral and central systems. Evidence obtained from patients and animal models of the disease gives clues of pathological alterations but does not as yet clearly answer this question.

Our current knowledge on pathological mechanisms involved in PD etiology and progression in humans is a consequence of the limited access to *in vivo* brain data. Cerebral imaging, while rapidly evolving in more powerful tools, has yet failed to give clear cut correlations between central dysfunctions and disease stage or progression, or with peripheral biological markers. Notwithstanding the development of improved radioligands for in vivo PET analyses, a clear understanding of the role of neuroinflammation in PD progression is still lacking. The comprehension of the complex nature of microglia cells, which can embrace a diversity of phenotype throughout the course of the disease, has guided de development of therapeutic strategies that would help mitigate their deleterious effect and modulate them toward a more “protective” phenotype rather them just inhibiting microglia activation. Exenatide, a GLP-1 receptor agonist, is a perfect example of a promising therapeutic alternative that may slow down disease progression through modulation of inflammation and neuroinflammation. GLP-1 receptors are highly expressed in microglia (237). In animals, exenatide has been shown to reduced MPTP-induced activation of microglia as well as the levels of inflammatory molecules including TNF-α and Il-β (238). Similar effects have been obtained in the rotenone model (239). The molecule, already available in the market for the treatment of insulin-resistant diabetes, has rapidly advanced in clinical trials for PD treatment. After a positive proof of concept open-label study that suggested significant improvement in both motor and non-motor PD features (240), researchers moved to a double-blind, placebo-controlled trial (241). Results indicate a significant improvement in motor scores in exenatide treated patients vs. the placebo group. Improvement in non-motor symptoms have also been reported for the same patients (242, 243). While the action of exenatide and how it may slow down PD progression is not clear, a recent evidence indicates that another GLP-1 agonist, NLY01, may limit microglia activation and reduce release of inflammatory molecules thus limiting neuroinflammation.

Preclinical models, in particular MPTP-treated mice, have also been used to evaluate the effect of minocycline, a tetracycline derivative, and showed that the molecule also possess anti-inflammatory properties, reducing microglia activation and Il-1β production in the SN (244). NET-PD FS 1, a futility trial in early untreated PD patients suggested that prolonged minocycline treatment caused no major safety concerns (245). Another antibiotic tetracycline molecule, doxycycline, has also shown promising results in animal models of PD, including 6-OHDA-treated mice (246), the LPS rat model (247). Doxycycline effect is associated with reduced microglia activation and may regulate inflammasome signaling (248). Today, large efforts are being made to modify the tetracycline molecules and separate their antimicrobial and anti-inflammatory properties to reduce any bacterial resistance that may occur after chronic use of antibiotics.

The inflammasome and its importance in neurodegenerative disease has attracted a lot of attention in the past years. All animal models of PD present upregulated levels of Il-1β both in peripheral and central compartment (Figure 3) and Il-1β is a major downstream activator of the inflammasome. Evidence indicates that blocking inflammasome with a small molecule called MCC950, which can readily cross the BBB, reduced Il-1β and caspase 1 levels and neurodegeneration in 6-OHDA animals and improved motor features in treated animals (249). MCC950 is being developed by a drug company that is hoping to start a Phase 1 clinical trial to determine the safety features in humans. Interestingly, many natural compounds with known anti-inflammatory properties have inhibitory activities on various components of the NLRP3 inflammasome pathway. Curcumin, resveratrol, and quercetin have all been shown to reduce levels of caspace-1 and Il-1β (250–252).

To date, most brain data still refer to postmortem pathology and thus only give a snapshot information, at a given and often advanced disease time point, on processes that may have been ongoing and have been evolving for years or decades. In the search for therapies that may slow down disease progression it is important to grasp the evolution of pathological mechanisms that are likely different at different disease stages. Current challenges and unmet needs in PD is the development of biomarkers that allow assessing ongoing dysregulated process in humans. Animal models largely contribute to the current knowledge of peripheral and central dysregulations that accompany neuronal cell loss. Numerous animal models have been developed in different organisms (25). While none of them fully recapitulate the multifactorial deficits observed in humans, they have, in particular, toxic transgenic rodent models, greatly helped address unanswered questions and are ideal for studying early and progressive neurodegenerative processes.

The perfect animal model that recapitulates all PD features does not exist. Yet animal models allow the evaluation of relevant genetic and environmental factors involved in PD pathology. Many immune alterations detected in PD patients have also been observed in animal models (see Figures 1–3). They have the advantage of presenting a homogeneous population, all individuals sharing identical genotype, and because they show high anatomical and physiological similarities to humans, they can be readily analyzed to understand the interrelationships and crosstalk between different body compartments. Importantly, animal models allow for relatively easy longitudinal studies. To date, few studies have assessed alterations in systemic compartments in available models. In the future, more systematic time-course evaluations of potential changes in peripheral immune factors are warranted. Similarly, the crossing of existing transgenic models or the combination of genetic and environmental factors in models to generate multiple hit triggers, will improve our knowledge on peripheral-central inflammation crosstalk.

Similarly, studies on patients have mostly concentrated on single point evaluation of immune alterations. Post-mortem studies give clear but static indication of late stage neuroinflammation alterations in PD brain, including infiltration of T or B lymphocytes infiltration or microglial activation. Similarly, analyses of levels of soluble factors and immune cells describe alterations at single time points. These studies have generated valuable information and have advanced our knowledge and understanding of PD. Yet, they represent a time point snapshot and barely or do not take into account the progressive nature of PD or the heterogeneity of the PD patient population. Longitudinal studies and patient stratification are desperately needed to gain a more comprehensive understanding of PD pathogenesis and disease progression.

In the past decades, biorepository resources and studies that allow collecting, processing, storing and distributing biospecimens have been developed to support research. These include the BioFIND study (completed in 2015), the Parkinson's Progression marker Initiative (PPMI), the Parkinson's Disease biomarker program (PDBP), the De Novo Parkinson (DeNoPa) in Germany, the ICEBERG study in France, the Norwegian Parkwest study, COPPADIS 2015 in Spain, the Oxford Parkinson's disease Centre and many more. These repositories aim at establishing a comprehensive follow up and collection of bio samples that will permit a better understanding of factors involved in disease progression, in particular in immune systems. The parallel development of both more precise imaging markers and more potent imaging equipment will allow a more precise *in vivo* quantification of neuroinflammatory processes, in particular microglial activation, that take place in affected brain structures, and allow correlation of ongoing immune alterations taking place in the brain and in peripheral systems.

## Conclusion

In the past decades, the development of animal models of PD has greatly contributed to expand our understanding of the disease. Animal models, transgenic, toxin- or viral induced, permit the analyses of more specific pathways and their impact on PD genotype. Today, the role of inflammation and neuroinflammation in the etiology and progression of PD, as well as the knowledge that both must closely interact is well-accepted. However, our understanding on how they communicate and combine to trigger and sustain neuronal cell death still needs to be refined. Comparing longitudinal data from patients and models will help us unravel the complicated mechanisms involved in peripheral-central inflammation crosstalk and open new ways of developing alternative therapeutic strategies to slow down disease progression.

## Data Availability

All datasets generated for this study are included in the manuscript and/or the supplementary files.

## Author Contributions

MF-A reviewed the literature and wrote the first draft of the manuscript. SC and FB reviewed the literature and critically reviewed the manuscript.

### Conflict of Interest Statement

The authors declare that the research was conducted in the absence of any commercial or financial relationships that could be construed as a potential conflict of interest.

## References

[1] Iwasaki A Medzhitov R. Control of adaptive immunity by the innate immune system. Nat Immunol. (2015) 16:343–53. 10.1038/ni.3123 25789684 PMC4507498

[2] Murphy K Innate Immunity: The First Line of Defense. Abingdon: Garland Science, Taylor & Francis group (2012).

[3] Janeway CA Jr. Approaching the asymptote? Evolution and revolution in immunology. Cold Spring Harb Symp Quant Biol. (1989) 54 (Pt. 1):1–13. 10.1101/SQB.1989.054.01.003 2700931

[4] Mosley RL. Adaptive immunity in neurodegenerative and neuropsychological disorders. J Neuroimmune Pharmacol. (2015) 10:522–7. 10.1007/s11481-015-9640-y 26496777

[5] Kannarkat GT Boss JM Tansey MG. The role of innate and adaptive immunity in Parkinson's disease. J Parkinsons Dis. (2013) 3:493–514. 10.3233/JPD-130250 24275605 PMC4102262

[6] Olsen L Dowd E Mckernan D A Role for viral infection in Parkinson's etiology–more than jsut “shaky” evidence? Neuronal Signal. (2018) 2:NS20180166 10.1042/NS20170166 PMC7373231 32714585

[7] Brundin P Melki R. Prying into the Prion Hypothesis for Parkinson's Disease. J Neurosci. (2017) 37:9808–18. 10.1523/JNEUROSCI.1788-16.2017 29021298 PMC5637113

[8] Xu L Pu J. Alpha-Synuclein in Parkinson's Disease: from pathogenetic dysfunction to potential clinical application. Parkinsons Dis. (2016) 2016:1720621. 10.1155/2016/1720621 27610264 PMC5005546

[9] George S Brundin P. Immunotherapy in Parkinson's Disease: micromanaging alpha-synuclein aggregation. J Parkinsons Dis. (2015) 5:413–24. 10.3233/JPD-150630 26406122 PMC4923719

[10] Allen Reish HE Standaert DG. Role of alpha-synuclein in inducing innate and adaptive immunity in Parkinson disease. J Parkinsons Dis. (2015) 5:1–19. 10.3233/JPD-140491 25588354 PMC4405142

[11] Galea I Bechmann I Perry VH. What is immune privilege (not)? Trends Immunol. (2007) 28:12–8. 10.1016/j.it.2006.11.004 17129764

[12] Li Q Barres BA. Microglia and macrophages in brain homeostasis and disease. Nat Rev Immunol. (2018) 18:225–42. 10.1038/nri.2017.125 29151590

[13] Tansey MG Goldberg MS. Neuroinflammation in Parkinson's disease: its role in neuronal death and implications for therapeutic intervention. Neurobiol Dis. (2010) 37:510–8. 10.1016/j.nbd.2009.11.004 19913097 PMC2823829

[14] Sochocka M Diniz BS Leszek J Inflammatory response in the CNS: friend or foe? Mol Neurobiol. (2017) 54:8071–89. 10.1007/s12035-016-0297 27889895 PMC5684251

[15] Disabato DJ Quan N Godbout JP. Neuroinflammation: the devil is in the details. J Neurochem. (2016) 139 (Suppl. 2):136–53. 10.1111/jnc.13607 26990767 PMC5025335

[16] Mcgeer PL Itagaki S Boyes BE Mcgeer EG. Reactive microglia are positive for HLA-DR in the substantia nigra of Parkinson's and Alzheimer's disease brains. Neurology. (1988) 38:1285–91. 10.1212/WNL.38.8.1285 3399080

[17] Mcgeer PL Itagaki S Mcgeer EG. Expression of the histocompatibility glycoprotein HLA-DR in neurological disease. Acta Neuropathol. (1988) 76:550–7. 10.1007/BF00689592 2974227

[18] Chen H Jacobs E Schwarzschild MA Mccullough ML Calle EE Thun MJ. Nonsteroidal antiinflammatory drug use and the risk for Parkinson's disease. Ann Neurol. (2005) 58:963–7. 10.1002/ana.20682 16240369

[19] Gagne JJ Power MC. Anti-inflammatory drugs and risk of Parkinson disease: a meta-analysis. Neurology. (2010) 74:995–1002. 10.1212/WNL.0b013e3181d5a4a3 20308684 PMC2848103

[20] Gao X Chen H Schwarzschild MA Ascherio A. Use of ibuprofen and risk of Parkinson disease. Neurology. (2011) 76:863–9. 10.1212/WNL.0b013e31820f2d79 21368281 PMC3059148

[21] Poly TN Islam MMR Yang HC Li YJ. Non-steroidal anti-inflammatory drugs and risk of Parkinson's disease in the elderly population: a meta-analysis. Eur J Clin Pharmacol. (2019) 75:99–108. 10.1007/s00228-018-2561-y 30280208

[22] Ren L Yi J Yang J Li P Cheng X Mao P. Nonsteroidal anti-inflammatory drugs use and risk of Parkinson disease: a dose-response meta-analysis. Medicine. (2018) 97:e12172. 10.1097/MD.0000000000012172 30212946 PMC6155958

[23] Bok E Cho EJ Chung ES Shin WH Jin BK. Interleukin-4 Contributes to Degeneration of Dopamine Neurons in the Lipopolysaccharide-treated Substantia Nigra *in vivo*. Exp Neurobiol. (2018) 27:309–19. 10.5607/en.2018.27.4.309 30181693 PMC6120964

[24] Spillantini MG Schmidt ML Lee VM Trojanowski JQ Jakes R Goedert M. Alpha-synuclein in Lewy bodies. Nature. (1997) 388:839–40. 10.1038/42166 9278044

[25] Breger LS Fuzzati Armentero MT. Genetically engineered animal models of Parkinson's disease: from worm to rodent. Eur J Neurosci. (2018) 49:533–60. 10.1111/ejn.14300 30552719

[26] Ajami B Bennett JL Krieger C Tetzlaff W Rossi FM. Local self-renewal can sustain CNS microglia maintenance and function throughout adult life. Nat Neurosci. (2007) 10:1538–43. 10.1038/nn2014 18026097

[27] Ginhoux F Greter M Leboeuf M Nandi S See P Gokhan S. Fate mapping analysis reveals that adult microglia derive from primitive macrophages. Science. (2010) 330:841–5. 10.1126/science.1194637 20966214 PMC3719181

[28] Askew K Li K Olmos-Alonso A Garcia-Moreno F Liang Y Richardson P. Coupled proliferation and apoptosis maintain the rapid turnover of microglia in the adult brain. Cell Rep. (2017) 18:391–405. 10.1016/j.celrep.2016.12.041 28076784 PMC5263237

[29] Tay TL Mai D Dautzenberg J Fernandez-Klett F Lin G Sagar Datta M. A new fate mapping system reveals context-dependent random or clonal expansion of microglia. Nat Neurosci. (2017) 20:793–803. 10.1038/nn.4547 28414331

[30] Davalos D Grutzendler J Yang G Kim JV Zuo Y Jung S. ATP mediates rapid microglial response to local brain injury *in vivo*. Nat Neurosci. (2005) 8:752–8. 10.1038/nn1472 15895084

[31] Nimmerjahn A Kirchhoff F Helmchen F. Resting microglial cells are highly dynamic surveillants of brain parenchyma *in vivo*. Science. (2005) 308:1314–8. 10.1126/science.1110647 15831717

[32] Wolf SA Boddeke HW Kettenmann H. Microglia in Physiology and Disease. Annu Rev Physiol. (2017) 79:619–43. 10.1146/annurev-physiol-022516-034406 27959620

[33] Deczkowska A Amit I Schwartz M Microglial immune checkpoint mechanisms. Nat Neurosci. (2018) 21:779–86. 10.1038/s41593-018-0145-x 29735982

[34] Hickman SE Kingery ND Ohsumi TK Borowsky ML Wang LC Means TK. The microglial sensome revealed by direct RNA sequencing. Nat Neurosci. (2013) 16:1896–905. 10.1038/nn.3554 24162652 PMC3840123

[35] Tay TL Savage JC Hui CW Bisht K Tremblay ME. Microglia across the lifespan: from origin to function in brain development, plasticity and cognition. J Physiol. (2017) 595:1929–45. 10.1113/JP272134 27104646 PMC5350449

[36] Joers V Tansey MG Mulas G Carta AR. Microglial phenotypes in Parkinson's disease and animal models of the disease. Prog Neurobiol. (2017) 155:57–75. 10.1016/j.pneurobio.2016.04.006 27107797 PMC5073045

[37] Imamura K Hishikawa N Sawada M Nagatsu T Yoshida M Hashizume Y. Distribution of major histocompatibility complex class II-positive microglia and cytokine profile of Parkinson's disease brains. Acta Neuropathol. (2003) 106:518–26. 10.1007/s00401-003-0766-2 14513261

[38] Doorn KJ Moors T Drukarch B Van De Berg W Lucassen PJ Van Dam AM. Microglial phenotypes and toll-like receptor 2 in the substantia nigra and hippocampus of incidental Lewy body disease cases and Parkinson's disease patients. Acta Neuropathol Commun. (2014) 2:90. 10.1186/s40478-014-0090-1 25099483 PMC4224021

[39] Hamza TH Zabetian CP Tenesa A Laederach A Montimurro J Yearout D. Common genetic variation in the HLA region is associated with late-onset sporadic Parkinson's disease. Nat Genet. (2010) 42:781–5. 10.1038/ng.642 20711177 PMC2930111

[40] Pierce S Coetzee GA. Parkinson's disease-associated genetic variation is linked to quantitative expression of inflammatory genes. PLoS ONE. (2017) 12:e0175882. 10.1371/journal.pone.0175882 28407015 PMC5391096

[41] Mondelli V Vernon AC Turkheimer F Dazzan P Pariante CM. Brain microglia in psychiatric disorders. Lancet Psychiatry. (2017) 4:563–72. 10.1016/S2215-0366(17)30101-3 28454915

[42] Gerhard A Pavese N Hotton G Turkheimer F Es M Hammers A *In vivo* imaging of microglial activation with [11C](R)-PK11195 PET in idiopathic Parkinson's disease. Neurobiol Dis. (2006) 21:404–12. 10.1016/j.nbd.2005.08.002 16182554

[43] Iannaccone S Cerami C Alessio M Garibotto V Panzacchi A Olivieri S. *In vivo* microglia activation in very early dementia with Lewy bodies, comparison with Parkinson's disease. Parkinsonism Relat Disord. (2013) 19:47–52. 10.1016/j.parkreldis.2012.07.002 22841687

[44] Ouchi Y Yoshikawa E Sekine Y Futatsubashi M Kanno T Ogusu T. Microglial activation and dopamine terminal loss in early Parkinson's disease. Ann Neurol. (2005) 57:168–75. 10.1002/ana.20338 15668962

[45] Owen DR Yeo AJ Gunn RN Song K Wadsworth G Lewis A. An 18-kDa translocator protein (TSPO) polymorphism explains differences in binding affinity of the PET radioligand PBR28. J Cereb Blood Flow Metab. (2012) 32:1–5. 10.1038/jcbfm.2011.147 22008728 PMC3323305

[46] Varnas K Cselenyi Z Jucaite A Halldin C Svenningsson P Farde L PET imaging of [(11)C]PBR28 in Parkinson's disease patients does not indicate increased binding to TSPO despite reduced dopamine transporter binding. Eur J Nucl Med Mol Imaging. (2019) 46:367–75. 10.1007/s00259-018-4161-6 30270409 PMC6333720

[47] Tsacopoulos M Magistretti PJ. Metabolic coupling between glia and neurons. J Neurosci. (1996) 16:877–85. 10.1523/JNEUROSCI.16-03-00877.1996 8558256 PMC6578818

[48] Mederos S Gonzalez-Arias C Perea G. Astrocyte-neuron networks: a multilane highway of signaling for homeostatic brain function. Front Synaptic Neurosci. (2018) 10:45. 10.3389/fnsyn.2018.00045 30542276 PMC6277918

[49] Braak H Sastre M Del Tredici K. Development of alpha-synuclein immunoreactive astrocytes in the forebrain parallels stages of intraneuronal pathology in sporadic Parkinson's disease. Acta Neuropathol. (2007) 114:231–41. 10.1007/s00401-007-0244-3 17576580

[50] Damier P Hirsch EC Zhang P Agid Y Javoy-Agid F. Glutathione peroxidase, glial cells and Parkinson's disease. Neuroscience. (1993) 52:1–6. 10.1016/0306-4522(93)90175-F 8433802

[51] Gray MT Woulfe JM. Striatal blood-brain barrier permeability in Parkinson's disease. J Cereb Blood Flow Metab. (2015) 35:747–50. 10.1038/jcbfm.2015.32 25757748 PMC4420870

[52] Ambrosi G Kustrimovic N Siani F Rasini E Cerri S Ghezzi C. Complex changes in the innate and adaptive immunity accompany progressive degeneration of the nigrostriatal pathway induced by intrastriatal injection of 6-hydroxydopamine in the rat. Neurotox Res. (2017) 32:71–81. 10.1007/s12640-017-9712-2 28285346

[53] Armentero MT Levandis G Nappi G Bazzini E Blandini F. Peripheral inflammation and neuroprotection: systemic pretreatment with complete Freund's adjuvant reduces 6-hydroxydopamine toxicity in a rodent model of Parkinson's disease. Neurobiol Dis. (2006) 24:492–505. 10.1016/j.nbd.2006.08.016 17023164

[54] Siani F Greco R Levandis G Ghezzi C Daviddi F Demartini C. Influence of estrogen modulation on glia activation in a murine model of Parkinson's Disease. Front Neurosci. (2017) 11:306. 10.3389/fnins.2017.00306 28620274 PMC5449471

[55] Dickens AM Vainio S Marjamaki P Johansson J Lehtiniemi P Rokka J. Detection of microglial activation in an acute model of neuroinflammation using PET and radiotracers 11C-(R)-PK11195 and 18F-GE-180. J Nucl Med. (2014) 55:466–72. 10.2967/jnumed.113.125625 24516258

[56] Vazquez-Claverie M Garrido-Gil P San Sebastian W Izal-Azcarate A Belzunegui S Marcilla I. Acute and chronic 1-methyl-4-phenyl-1,2,3,6-tetrahydropyridine administrations elicit similar microglial activation in the substantia nigra of monkeys. J Neuropathol Exp Neurol. (2009) 68:977–84. 10.1097/NEN.0b013e3181b35e41 19680145

[57] Belloli S Pannese M Buonsanti C Maiorino C Di Grigoli G Carpinelli A. Early upregulation of 18-kDa translocator protein in response to acute neurodegenerative damage in TREM2-deficient mice. Neurobiol Aging. (2017) 53:159–68. 10.1016/j.neurobiolaging.2017.01.010 28189343

[58] Pan-Montojo F Anichtchik O Dening Y Knels L Pursche S Jung R. Progression of Parkinson's disease pathology is reproduced by intragastric administration of rotenone in mice. PLoS ONE. (2010) 5:e8762. 10.1371/journal.pone.0008762 20098733 PMC2808242

[59] Pan-Montojo F Funk RH. Implications of Parkinson's disease pathophysiology for the development of cell replacement strategies and drug discovery in neurodegenerative diseases. CNS Neurol Disord Drug Targets. (2012) 11:907–20. 10.2174/1871527311201070907 23131153

[60] Sherer TB Kim JH Betarbet R Greenamyre JT. Subcutaneous rotenone exposure causes highly selective dopaminergic degeneration and alpha-synuclein aggregation. Exp Neurol. (2003) 179:9–16. 10.1006/exnr.2002.8072 12504863

[61] Marinova-Mutafchieva L Sadeghian M Broom L Davis JB Medhurst AD Dexter DT. Relationship between microglial activation and dopaminergic neuronal loss in the substantia nigra: a time course study in a 6-hydroxydopamine model of Parkinson's disease. J Neurochem. (2009) 110:966–75. 10.1111/j.1471-4159.2009.06189.x 19549006

[62] Cicchetti F Brownell AL Williams K Chen YI Livni E Isacson O. Neuroinflammation of the nigrostriatal pathway during progressive 6-OHDA dopamine degeneration in rats monitored by immunohistochemistry and PET imaging. Eur J Neurosci. (2002) 15:991–8. 10.1046/j.1460-9568.2002.01938.x 11918659

[63] Mcgeer PL Schwab C Parent A Doudet D. Presence of reactive microglia in monkey substantia nigra years after 1-methyl-4-phenyl-1,2,3,6-tetrahydropyridine administration. Ann Neurol. (2003) 54:599–604. 10.1002/ana.10728 14595649

[64] Schintu N Frau L Ibba M Garau A Carboni E Carta AR. Progressive dopaminergic degeneration in the chronic MPTPp mouse model of Parkinson's disease. Neurotox Res. (2009) 16:127–39. 10.1007/s12640-009-9061-x 19526289

[65] Yasuda Y Shinagawa R Yamada M Mori T Tateishi N Fujita S. Long-lasting reactive changes observed in microglia in the striatal and substantia nigral of mice after 1-methyl-4-phenyl-1,2,3,6-tetrahydropyridine. Brain Res. (2007) 1138:196–202. 10.1016/j.brainres.2006.12.054 17275793

[66] Kurkowska-Jastrzebska I Wronska A Kohutnicka M Czlonkowski A Czlonkowska A. The inflammatory reaction following 1-methyl-4-phenyl-1,2,3,6–tetrahydropyridine intoxication in mouse. Exp Neurol. (1999) 156:50–61. 10.1006/exnr.1998.6993 10192776

[67] Pisanu A Lecca D Mulas G Wardas J Simbula G Spiga S. Dynamic changes in pro- and anti-inflammatory cytokines in microglia after PPAR-gamma agonist neuroprotective treatment in the MPTPp mouse model of progressive Parkinson's disease. Neurobiol Dis. (2014) 71:280–91. 10.1016/j.nbd.2014.08.011 25134730

[68] Betarbet R Sherer TB Mackenzie G Garcia-Osuna M Panov AV Greenamyre JT. Chronic systemic pesticide exposure reproduces features of Parkinson's disease. Nat Neurosci. (2000) 3:1301–6. 10.1038/81834 11100151

[69] Cicchetti F Drouin-Ouellet J Gross RE. Environmental toxins and Parkinson's disease: what have we learned from pesticide-induced animal models? Trends Pharmacol Sci. (2009) 30:475–83. 10.1016/j.tips.2009.06.005 19729209

[70] Ojha S Javed H Azimullah S Abul Khair SB Haque ME. Neuroprotective potential of ferulic acid in the rotenone model of Parkinson's disease. Drug Des Devel Ther. (2015) 9:5499–510. 10.2147/DDDT.S90616 26504373 PMC4603721

[71] Dutta G Zhang P Liu B. The lipopolysaccharide Parkinson's disease animal model: mechanistic studies and drug discovery. Fundam Clin Pharmacol. (2008) 22:453–64. 10.1111/j.1472-8206.2008.00616.x 18710400 PMC2632601

[72] Thomas RC Bath MF Stover CM Lambert DG Thompson JP. Exploring LPS-induced sepsis in rats and mice as a model to study potential protective effects of the nociceptin/orphanin FQ system. Peptides. (2014) 61:56–60. 10.1016/j.peptides.2014.08.009 25161013

[73] Hauss-Wegrzyniak B Lukovic L Bigaud M Stoeckel ME. Brain inflammatory response induced by intracerebroventricular infusion of lipopolysaccharide: an immunohistochemical study. Brain Res. (1998) 794:211–24. 10.1016/S0006-8993(98)00227-3 9622633

[74] Liu M Bing G. Lipopolysaccharide animal models for Parkinson's disease. Parkinsons Dis. (2011) 2011:327089. 10.4061/2011/327089 21603177 PMC3096023

[75] Beier EE Neal M Alam G Edler M Wu LJ Richardson JR. Alternative microglial activation is associated with cessation of progressive dopamine neuron loss in mice systemically administered lipopolysaccharide. Neurobiol Dis. (2017) 108:115–27. 10.1016/j.nbd.2017.08.009 28823928 PMC5734673

[76] Su X Maguire-Zeiss KA Giuliano R Prifti L Venkatesh K Federoff HJ. Synuclein activates microglia in a model of Parkinson's disease. Neurobiol Aging. (2008) 29:1690–701. 10.1016/j.neurobiolaging.2007.04.006 17537546 PMC2621109

[77] Watson MB Richter F Lee SK Gabby L Wu J Masliah E. Regionally-specific microglial activation in young mice over-expressing human wildtype alpha-synuclein. Exp Neurol. (2012) 237:318–34. 10.1016/j.expneurol.2012.06.025 22750327 PMC3443323

[78] Volpicelli-Daley LA Kirik D Stoyka LE Standaert DG Harms AS. How can rAAV-alpha-synuclein and the fibril alpha-synuclein models advance our understanding of Parkinson's disease? J Neurochem. (2016) 139 (Suppl. 1):131–55. 10.1111/jnc.13627 27018978 PMC5040622

[79] Eslamboli A Romero-Ramos M Burger C Bjorklund T Muzyczka N Mandel RJ. Long-term consequences of human alpha-synuclein overexpression in the primate ventral midbrain. Brain. (2007) 130:799–815. 10.1093/brain/awl382 17303591

[80] Harms AS Cao S Rowse AL Thome AD Li X Mangieri LR. MHCII is required for alpha-synuclein-induced activation of microglia, CD4 T cell proliferation, and dopaminergic neurodegeneration. J Neurosci. (2013) 33:9592–600. 10.1523/JNEUROSCI.5610-12.2013 23739956 PMC3903980

[81] Sanchez-Guajardo V Febbraro F Kirik D Romero-Ramos M. Microglia acquire distinct activation profiles depending on the degree of alpha-synuclein neuropathology in a rAAV based model of Parkinson's disease. PLoS ONE. (2010) 5:e8784. 10.1371/journal.pone.0008784 20098715 PMC2808388

[82] Theodore S Cao S Mclean PJ Standaert DG. Targeted overexpression of human alpha-synuclein triggers microglial activation and an adaptive immune response in a mouse model of Parkinson disease. J Neuropathol Exp Neurol. (2008) 67:1149–58. 10.1097/NEN.0b013e31818e5e99 19018246 PMC2753200

[83] Barkholt P Sanchez-Guajardo V Kirik D Romero-Ramos M. Long-term polarization of microglia upon alpha-synuclein overexpression in nonhuman primates. Neuroscience. (2012) 208:85–96. 10.1016/j.neuroscience.2012.02.004 22342967

[84] Ferreira SA Romero-Ramos M. Microglia Response During Parkinson's Disease: alpha-synuclein intervention. Front Cell Neurosci. (2018) 12:247. 10.3389/fncel.2018.00247 30127724 PMC6087878

[85] Shechter R London A Schwartz M. Orchestrated leukocyte recruitment to immune-privileged sites: absolute barriers versus educational gates. Nat Rev Immunol. (2013) 13:206–18. 10.1038/nri3391 23435332

[86] Cabezas R Avila M Gonzalez J El-Bacha RS Baez E Garcia-Segura LM. Astrocytic modulation of blood brain barrier: perspectives on Parkinson's disease. Front Cell Neurosci. (2014) 8:211. 10.3389/fncel.2014.00211 25136294 PMC4120694

[87] Zlokovic BV. The blood-brain barrier in health and chronic neurodegenerative disorders. Neuron. (2008) 57:178–201. 10.1016/j.neuron.2008.01.003 18215617

[88] Goldmann T Wieghofer P Jordao MJ Prutek F Hagemeyer N Frenzel K. Origin, fate and dynamics of macrophages at central nervous system interfaces. Nat Immunol. (2016) 17:797–805. 10.1038/ni.3423 27135602 PMC4968048

[89] Falcao AM Marques F Novais A Sousa N Palha JA Sousa JC. The path from the choroid plexus to the subventricular zone: go with the flow! Front Cell Neurosci. (2012) 6:34. 10.3389/fncel.2012.00034 22907990 PMC3414909

[90] Emerich DF Skinner SJ Borlongan CV Vasconcellos AV Thanos CG. The choroid plexus in the rise, fall and repair of the brain. Bioessays. (2005) 27:262–74. 10.1002/bies.20193 15714561

[91] Johanson CE Stopa EG Mcmillan PN. The blood-cerebrospinal fluid barrier: structure and functional significance. Methods Mol Biol. (2011) 686:101–31. 10.1007/978-1-60761-938-3_4 21082368

[92] Svenningsson A Andersen O Edsbagge M Stemme S. Lymphocyte phenotype and subset distribution in normal cerebrospinal fluid. J Neuroimmunol. (1995) 63:39–46. 10.1016/0165-5728(95)00126-3 8557823

[93] Derecki NC Cardani AN Yang CH Quinnies KM Crihfield A Lynch KR. Regulation of learning and memory by meningeal immunity: a key role for IL-4. J Exp Med. (2010) 207:1067–80. 10.1084/jem.20091419 20439540 PMC2867291

[94] Benakis C Llovera G Liesz A. The meningeal and choroidal infiltration routes for leukocytes in stroke. Ther Adv Neurol Disord. (2018) 11:1756286418783708. 10.1177/1756286418783708 29977343 PMC6024265

[95] Herz J Filiano AJ Smith A Yogev N Kipnis J. Myeloid cells in the central nervous system. Immunity. (2017) 46:943–56. 10.1016/j.immuni.2017.06.007 28636961 PMC5657250

[96] Korin B Ben-Shaanan TL Schiller M Dubovik T Azulay-Debby H Boshnak NT. High-dimensional, single-cell characterization of the brain's immune compartment. Nat Neurosci. (2017) 20:1300–9. 10.1038/nn.4610 28758994

[97] Kortekaas R Leenders KL Van Oostrom JC Vaalburg W Bart J Willemsen AT. Blood-brain barrier dysfunction in Parkinsonian midbrain *in vivo*. Ann Neurol. (2005) 57:176–9. 10.1002/ana.20369 15668963

[98] Bartels AL Willemsen AT Kortekaas R De Jong BM De Vries R De Klerk O. Decreased blood-brain barrier P-glycoprotein function in the progression of Parkinson's disease, PSP and MSA. J Neural Transm. (2008) 115:1001–9. 10.1007/s00702-008-0030-y 18265929 PMC2468317

[99] Pienaar IS Lee CH Elson JL Mcguinness L Gentleman SM Kalaria RN. Deep-brain stimulation associates with improved microvascular integrity in the subthalamic nucleus in Parkinson's disease. Neurobiol Dis. (2015) 74:392–405. 10.1016/j.nbd.2014.12.006 25533682

[100] Desai Bradaric B Patel A Schneider JA Carvey PM Hendey B. Evidence for angiogenesis in Parkinson's disease, incidental Lewy body disease, and progressive supranuclear palsy. J Neural Transm. (2012) 119:59–71. 10.1007/s00702-011-0684-8 21748523 PMC3352316

[101] Wada K Arai H Takanashi M Fukae J Oizumi H Yasuda T. Expression levels of vascular endothelial growth factor and its receptors in Parkinson's disease. Neuroreport. (2006) 17:705–9. 10.1097/01.wnr.0000215769.71657.65 16641673

[102] Janelidze S Lindqvist D Francardo V Hall S Zetterberg H Blennow K. Increased CSF biomarkers of angiogenesis in Parkinson disease. Neurology. (2015) 85:1834–42. 10.1212/WNL.0000000000002151 26511451 PMC4662706

[103] Pisani V Stefani A Pierantozzi M Natoli S Stanzione P Franciotta D. Increased blood-cerebrospinal fluid transfer of albumin in advanced Parkinson's disease. J Neuroinflammation. (2012) 9:188. 10.1186/1742-2094-9-188 22870899 PMC3441323

[104] Al-Bachari S Vidyasagar R Emsley HC Parkes LM. Structural and physiological neurovascular changes in idiopathic Parkinson's disease and its clinical phenotypes. J Cereb Blood Flow Metab. (2017) 37:3409–21. 10.1177/0271678X16688919 28112022 PMC5624390

[105] Ham JH Yi H Sunwoo MK Hong JY Sohn YH Lee PH. Cerebral microbleeds in patients with Parkinson's disease. J Neurol. (2014) 261:1628–35. 10.1007/s00415-014-7403-y 24920492

[106] Garcia-Dominguez I Vesela K Garcia-Revilla J Carrillo-Jimenez A Roca-Ceballos MA Santiago M. Peripheral inflammation enhances microglia response and nigral dopaminergic cell death in an *in vivo* MPTP Model of Parkinson's Disease. Front Cell Neurosci. (2018) 12:398. 10.3389/fncel.2018.00398 30459561 PMC6232526

[107] Olmedo-Diaz S Estevez-Silva H Oradd G Af Bjerken S Marcellino D Virel A. An altered blood-brain barrier contributes to brain iron accumulation and neuroinflammation in the 6-OHDA rat model of Parkinson's disease. Neuroscience. (2017) 362:141–51. 10.1016/j.neuroscience.2017.08.023 28842186

[108] Armentero MT Levandis G Bazzini E Cerri S Ghezzi C Blandini F. Adhesion molecules as potential targets for neuroprotection in a rodent model of Parkinson's disease. Neurobiol Dis. (2011) 43:663–8. 10.1016/j.nbd.2011.05.017 21684338

[109] Carvey PM Zhao CH Hendey B Lum H Trachtenberg J Desai BS. 6-Hydroxydopamine-induced alterations in blood-brain barrier permeability. Eur J Neurosci. (2005) 22:1158–68. 10.1111/j.1460-9568.2005.04281.x 16176358

[110] Brochard V Combadiere B Prigent A Laouar Y Perrin A Beray-Berthat V. Infiltration of CD4^+^ lymphocytes into the brain contributes to neurodegeneration in a mouse model of Parkinson disease. J Clin Invest. (2009) 119:182–92. 10.1172/JCI36470 19104149 PMC2613467

[111] Miklossy J Doudet DD Schwab C Yu S Mcgeer EG Mcgeer PL. Role of ICAM-1 in persisting inflammation in Parkinson disease and MPTP monkeys. Exp Neurol. (2006) 197:275–83. 10.1016/j.expneurol.2005.10.034 16336966

[112] Barcia C Bautista V Sanchez-Bahillo A Fernandez-Villalba E Faucheux B Poza Y Poza M. Changes in vascularization in substantia nigra pars compacta of monkeys rendered Parkinsonian. J Neural Transm. (2005) 112:1237–48. 10.1007/s00702-004-0256-2 15666038

[113] Thiollier T Wu C Contamin H Li Q Zhang J Bezard E. Permeability of blood-brain barrier in macaque model of 1-methyl-4-phenyl-1,2,3,6-tetrahydropyridine-induced Parkinson disease. Synapse. (2016) 70:231–9. 10.1002/syn.21889 26799359

[114] Chung YC Shin WH Baek JY Cho EJ Baik HH Kim SR. CB2 receptor activation prevents glial-derived neurotoxic mediator production, BBB leakage and peripheral immune cell infiltration and rescues dopamine neurons in the MPTP model of Parkinson's disease. Exp Mol Med. (2016) 48:e205. 10.1038/emm.2015.100 27534533 PMC4892852

[115] Ravenstijn PG Merlini M Hameetman M Murray TK Ward MA Lewis H. The exploration of rotenone as a toxin for inducing Parkinson's disease in rats, for application in BBB transport and PK-PD experiments. J Pharmacol Toxicol Methods. (2008) 57:114–30. 10.1016/j.vascn.2007.10.003 18155613

[116] Banks WA. Blood-brain barrier transport of cytokines: a mechanism for neuropathology. Curr Pharm Des. (2005) 11:973–84. 10.2174/1381612053381684 15777248

[117] Hunot S Dugas N Faucheux B Hartmann A Tardieu M Debre P. FcepsilonRII/CD23 is expressed in Parkinson's disease and induces, *in vitro*, production of nitric oxide and tumor necrosis factor-alpha in glial cells. J Neurosci. (1999) 19:3440–7. 10.1523/JNEUROSCI.19-09-03440.1999 10212304 PMC6782235

[118] Mogi M Harada M Narabayashi H Inagaki H Minami M Nagatsu T. Interleukin (IL)-1 beta, IL-2, IL-4, IL-6 and transforming growth factor-alpha levels are elevated in ventricular cerebrospinal fluid in juvenile parkinsonism and Parkinson's disease. Neurosci Lett. (1996) 211:13–6. 10.1016/0304-3940(96)12706-3 8809836

[119] Mogi M Harada M Kondo T Narabayashi H Riederer P Nagatsu T. Transforming growth factor-beta 1 levels are elevated in the striatum and in ventricular cerebrospinal fluid in Parkinson's disease. Neurosci Lett. (1995) 193:129–32. 10.1016/0304-3940(95)11686-Q 7478158

[120] Mogi M Harada M Riederer P Narabayashi H Fujita K Nagatsu T. Tumor necrosis factor-alpha (TNF-alpha) increases both in the brain and in the cerebrospinal fluid from Parkinsonian patients. Neurosci Lett. (1994) 165:208–10. 10.1016/0304-3940(94)90746-3 8015728

[121] Shimoji M Pagan F Healton EB Mocchetti I. CXCR4 and CXCL12 expression is increased in the nigro-striatal system of Parkinson's disease. Neurotox Res. (2009) 16:318–28. 10.1007/s12640-009-9076-3 19551455

[122] Brodacki B Staszewski J Toczylowska B Kozlowska E Drela N Chalimoniuk M. Serum interleukin (IL-2, IL-10, IL-6, IL-4), TNFalpha, and INFgamma concentrations are elevated in patients with atypical and idiopathic parkinsonism. Neurosci Lett. (2008) 441:158–62. 10.1016/j.neulet.2008.06.040 18582534

[123] Choi C Jeong JH Jang JS Choi K Lee J Kwon J. Multiplex analysis of cytokines in the serum and cerebrospinal fluid of patients with Alzheimer's disease by color-coded bead technology. J Clin Neurol. (2008) 4:84–8. 10.3988/jcn.2008.4.2.84 19513308 PMC2686871

[124] Dursun E Gezen-Ak D Hanagasi H Bilgic B Lohmann E Ertan S The interleukin 1 alpha, interleukin 1 beta, interleukin 6 and alpha-2-macroglobulin serum levels in patients with early or late onset Alzheimer's disease, mild cognitive impairment or Parkinson's disease. J Neuroimmunol. (2015) 283:50–7. 10.1016/j.jneuroim.2015.04.014 26004156

[125] Hall S Janelidze S Surova Y Widner H Zetterberg H Hansson O. Cerebrospinal fluid concentrations of inflammatory markers in Parkinson's disease and atypical Parkinsonian disorders. Sci Rep. (2018) 8:13276. 10.1038/s41598-018-31517-z 30185816 PMC6125576

[126] Williams-Gray CH Wijeyekoon R Yarnall AJ Lawson RA Breen DP Evans JR. Serum immune markers and disease progression in an incident Parkinson's disease cohort (ICICLE-PD). Mov Disord. (2016) 31:995–1003. 10.1002/mds.26563 26999434 PMC4957620

[127] Qin XY Zhang SP Cao C Loh YP Cheng Y. Aberrations in Peripheral Inflammatory Cytokine Levels in Parkinson Disease: a systematic review and meta-analysis. JAMA Neurol. (2016) 73:1316–24. 10.1001/jamaneurol.2016.2742 27668667

[128] Eidson LN Kannarkat GT Barnum CJ Chang J Chung J Caspell-Garcia C. Candidate inflammatory biomarkers display unique relationships with alpha-synuclein and correlate with measures of disease severity in subjects with Parkinson's disease. J Neuroinflammation. (2017) 14:164. 10.1186/s12974-017-0935-1 28821274 PMC5563061

[129] Karpenko MN Vasilishina AA Gromova EA Muruzheva ZM Bernadotte A. Interleukin-1beta, interleukin-1 receptor antagonist, interleukin-6, interleukin-10, and tumor necrosis factor-alpha levels in CSF and serum in relation to the clinical diversity of Parkinson's disease. Cell Immunol. (2018) 327:77–82. 10.1016/j.cellimm.2018.02.011 29478949

[130] Tang P Chong L Li X Liu Y Liu P Hou C. Correlation between serum RANTES levels and the severity of Parkinson's disease. Oxid Med Cell Longev. (2014) 2014:208408. 10.1155/2014/208408 25587378 PMC4283268

[131] Rocha NP Scalzo PL Barbosa IG Souza MS Morato IB Vieira EL. Cognitive status correlates with CXCL10/IP-10 Levels in Parkinson's Disease. Parkinsons Dis. (2014) 2014:903796. 10.1155/2014/903796 25386381 PMC4216701

[132] Linquist S Saylor B Cottenie K Elliott TA Kremer SC Gregory TR. Distinguishing ecological from evolutionary approaches to transposable elements. Biol Rev Camb Philos Soc. (2013) 88:573–84. 10.1111/brv.12017 23347261

[133] Shi M Bradner J Hancock AM Chung KA Quinn JF Peskind ER. Cerebrospinal fluid biomarkers for Parkinson disease diagnosis and progression. Ann Neurol. (2011) 69:570–80. 10.1002/ana.22311 21400565 PMC3117674

[134] Schroder JB Pawlowski M Meyer Zu Horste G Gross CC Wiendl H Meuth SG. Immune cell activation in the cerebrospinal fluid of patients with Parkinson's Disease. Front Neurol. (2018) 9:1081. 10.3389/fneur.2018.01081 30619041 PMC6305582

[135] Mogi M Harada M Kondo T Riederer P Inagaki H Minami M. Interleukin-1 beta, interleukin-6, epidermal growth factor and transforming growth factor-alpha are elevated in the brain from parkinsonian patients. Neurosci Lett. (1994) 180:147–50. 10.1016/0304-3940(94)90508-8 7700568

[136] Boka G Anglade P Wallach D Javoy-Agid F Agid Y Hirsch EC. Immunocytochemical analysis of tumor necrosis factor and its receptors in Parkinson's disease. Neurosci Lett. (1994) 172:151–4. 10.1016/0304-3940(94)90684-X 8084523

[137] McCoy MK Martinez TN Ruhn KA Szymkowski DE Smith CG Botterman BR. Blocking soluble tumor necrosis factor signaling with dominant-negative tumor necrosis factor inhibitor attenuates loss of dopaminergic neurons in models of Parkinson's disease. J Neurosci. (2006) 26:9365–75. 10.1523/JNEUROSCI.1504-06.2006 16971520 PMC3707118

[138] Pott Godoy MC Tarelli R Ferrari CC Sarchi MI Pitossi FJ. Central and systemic IL-1 exacerbates neurodegeneration and motor symptoms in a model of Parkinson's disease. Brain. (2008) 131:1880–94. 10.1093/brain/awn101 18504291 PMC2442423

[139] Koprich JB Reske-Nielsen C Mithal P Isacson O. Neuroinflammation mediated by IL-1beta increases susceptibility of dopamine neurons to degeneration in an animal model of Parkinson's disease. J Neuroinflammation. (2008) 5:8. 10.1186/1742-2094-5-8 18304357 PMC2292163

[140] Chien CH Lee MJ Liou HC Liou HH Fu WM. Microglia-Derived Cytokines/Chemokines Are Involved in the Enhancement of LPS-Induced Loss of Nigrostriatal Dopaminergic Neurons in DJ-1 Knockout Mice. PLoS ONE. (2016) 11:e0151569. 10.1371/journal.pone.0151569 26982707 PMC4794203

[141] Frank-Cannon TC Tran T Ruhn KA Martinez TN Hong J Marvin M. Parkin deficiency increases vulnerability to inflammation-related nigral degeneration. J Neurosci. (2008) 28:10825–34. 10.1523/JNEUROSCI.3001-08.2008 18945890 PMC2603252

[142] Depino AM Earl C Kaczmarczyk E Ferrari C Besedovsky H Del Rey A. Microglial activation with atypical proinflammatory cytokine expression in a rat model of Parkinson's disease. Eur J Neurosci. (2003) 18:2731–42. 10.1111/j.1460-9568.2003.03014.x 14656322

[143] Vairetti M Ferrigno A Rizzo V Ambrosi G Bianchi A Richelmi P. Impaired hepatic function and central dopaminergic denervation in a rodent model of Parkinson's disease: a self-perpetuating crosstalk? Biochim Biophys Acta. (2012) 1822:176–84. 10.1016/j.bbadis.2011.11.008 22119596

[144] Barcia C Ros CM Annese V Gomez A Ros-Bernal F Aguado-Yera D. IFN-gamma signaling, with the synergistic contribution of TNF-alpha, mediates cell specific microglial and astroglial activation in experimental models of Parkinson's disease. Cell Death Dis. (2011) 2:e142. 10.1038/cddis.2011.17 21472005 PMC3122054

[145] Nagatsu T Mogi M Ichinose H Togari A. Changes in cytokines and neurotrophins in Parkinson's disease. J Neural Transm Suppl. (2000) 277–90. 10.1007/978-3-7091-6301-6_19 11205147

[146] Sriram K Matheson JM Benkovic SA Miller DB Luster MI O'callaghan JP. Mice deficient in TNF receptors are protected against dopaminergic neurotoxicity: implications for Parkinson's disease. FASEB J. (2002) 16:1474–6. 10.1096/fj.02-0216fje 12205053

[147] Mount MP Lira A Grimes D Smith PD Faucher S Slack R. Involvement of interferon-gamma in microglial-mediated loss of dopaminergic neurons. J Neurosci. (2007) 27:3328–37. 10.1523/JNEUROSCI.5321-06.2007 17376993 PMC6672486

[148] Barcia C Ros CM Annese V Gomez A Ros-Bernal F Aguado-Llera D. IFN-gamma signaling, with the synergistic contribution of TNF-alpha, mediates cell specific microglial and astroglial activation in experimental models of Parkinson's disease. Cell Death Dis. (2012) 3:e379. 10.1038/cddis.2012.123 22914327 PMC3434670

[149] Bian MJ Li LM Yu M Fei J Huang F. Elevated interleukin-1beta induced by 1-methyl-4-phenyl-1,2,3,6-tetrahydropyridine aggravating dopaminergic neurodegeneration in old male mice. Brain Res. (2009) 1302:256–64. 10.1016/j.brainres.2009.07.030 19631617

[150] Lofrumento DD Saponaro C Cianciulli A De Nuccio F Mitolo V Nicolardi G. MPTP-induced neuroinflammation increases the expression of pro-inflammatory cytokines and their receptors in mouse brain. Neuroimmunomodulation. (2011) 18:79–88. 10.1159/000320027 20938211

[151] Yasuda Y Shimoda T Uno K Tateishi N Furuya S Yagi K. The effects of MPTP on the activation of microglia/astrocytes and cytokine/chemokine levels in different mice strains. J Neuroimmunol. (2008) 204:43–51. 10.1016/j.jneuroim.2008.08.003 18817984

[152] Luchtman DW Shao D Song C. Behavior, neurotransmitters and inflammation in three regimens of the MPTP mouse model of Parkinson's disease. Physiol Behav. (2009) 98:130–8. 10.1016/j.physbeh.2009.04.021 19410592

[153] Zhou T Zhu M Liang Z. (-)-Epigallocatechin-3-gallate modulates peripheral immunity in the MPTP-induced mouse model of Parkinson's disease. Mol Med Rep. (2018) 17:4883–8. 10.3892/mmr.2018.8470 29363729 PMC5865947

[154] Chandra G Rangasamy SB Roy A Kordower JH Pahan K. Neutralization of RANTES and eotaxin prevents the loss of dopaminergic neurons in a mouse model of Parkinson Disease. J Biol Chem. (2016) 291:15267–81. 10.1074/jbc.M116.714824 27226559 PMC4946939

[155] Chandra G Roy A Rangasamy SB Pahan K. Induction of adaptive immunity leads to nigrostriatal disease progression in MPTP Mouse Model of Parkinson's Disease. J Immunol. (2017) 198:4312–26. 10.4049/jimmunol.1700149 28446566 PMC5467696

[156] Appay V Rowland-Jones SL. RANTES: a versatile and controversial chemokine. Trends Immunol. (2001) 22:83–7. 10.1016/S1471-4906(00)01812-3 11286708

[157] Wada T Furuichi K Sakai N Shimizu M Segawa C Kobayashi K. Eotaxin contributes to renal interstitial eosinophilia. Nephrol Dial Transplant. (1999) 14:76–80. 10.1093/ndt/14.1.76 10052481

[158] Roy A Mondal S Kordower JH Pahan K. Attenuation of microglial RANTES by NEMO-binding domain peptide inhibits the infiltration of CD8(+) T cells in the nigra of Hemiparkinsonian monkey. Neuroscience. (2015) 302:36–46. 10.1016/j.neuroscience.2015.03.011 25783477 PMC4527882

[159] Roy A Fung YK Liu X Pahan K. Up-regulation of microglial CD11b expression by nitric oxide. J Biol Chem. (2006) 281:14971–80. 10.1074/jbc.M600236200 16551637 PMC1963414

[160] Roy A Jana A Yatish K Freidt MB Fung YK Martinson JA. Reactive oxygen species up-regulate CD11b in microglia via nitric oxide: Implications for neurodegenerative diseases. Free Radic Biol Med. (2008) 45:686–99. 10.1016/j.freeradbiomed.2008.05.026 18590811 PMC2701551

[161] Perry VH Holmes C. Microglial priming in neurodegenerative disease. Nat Rev Neurol. (2014) 10:217–24. 10.1038/nrneurol.2014.38 24638131

[162] Perry VH. Stress primes microglia to the presence of systemic inflammation: implications for environmental influences on the brain. Brain Behav Immun. (2007) 21:45–6. 10.1016/j.bbi.2006.08.004 17011745

[163] Cunningham C Campion S Lunnon K Murray CL Woods JF Deacon RM. Systemic inflammation induces acute behavioral and cognitive changes and accelerates neurodegenerative disease. Biol Psychiatry. (2009) 65:304–12. 10.1016/j.biopsych.2008.07.024 18801476 PMC2633437

[164] Dasgupta S Jana M Liu X Pahan K. Role of very-late antigen-4 (VLA-4) in myelin basic protein-primed T cell contact-induced expression of proinflammatory cytokines in microglial cells. J Biol Chem. (2003) 278:22424–31. 10.1074/jbc.M301789200 12690109 PMC1955481

[165] Dasgupta S Jana M Liu X Pahan K Myelin basic protein-primed T cells of female but not male mice induce nitric-oxide synthase and proinflammatory cytokines in microglia: implications for gender bias in multiple sclerosis. J Biol Chem. (2005) 280:32609–17. 10.1074/jbc.M500299200 16046404 PMC1955478

[166] Jana M Pahan K Induction of lymphotoxin-alpha by interleukin-12 p40 homodimer, the so-called biologically inactive molecule, but not IL-12 p70. Immunology. (2009) 127:312–25. 10.1111/j.1365-2567.2008.02985.x 19019087 PMC2712100

[167] Jiang S Gao H Luo Q Wang P Yang X. The correlation of lymphocyte subsets, natural killer cell, and Parkinson's disease: a meta-analysis. Neurol Sci. (2017) 38:1373–80. 10.1007/s10072-017-2988-4 28497309

[168] Kustrimovic N Comi C Magistrelli L Rasini E Legnaro M Bombelli R. Parkinson's disease patients have a complex phenotypic and functional Th1 bias: cross-sectional studies of CD4+ Th1/Th2/T17 and Treg in drug-naive and drug-treated patients. J Neuroinflammation. (2018) 15:205. 10.1186/s12974-018-1248-8 30001736 PMC6044047

[169] Saunders JA Estes KA Kosloski LM Allen HE Dempsey KM Torres-Russotto DR. CD4^+^ regulatory and effector/memory T cell subsets profile motor dysfunction in Parkinson's disease. J Neuroimmune Pharmacol. (2012) 7:927–38. 10.1007/s11481-012-9402-z 23054369 PMC3515774

[170] Levite M. Dopamine and T cells: dopamine receptors and potent effects on T cells, dopamine production in T cells, and abnormalities in the dopaminergic system in T cells in autoimmune, neurological and psychiatric diseases. Acta Physiol. (2016) 216:42–89. 10.1111/apha.12476 25728499

[171] Martinez EM Young AL Patankar YR Berwin BL Wang L Von Herrmann KM. Editor's Highlight: Nlrp3 Is required for inflammatory changes and nigral cell loss resulting from chronic intragastric rotenone exposure in mice. Toxicol Sci. (2017) 159:64–75. 10.1093/toxsci/kfx117 28903492 PMC5837210

[172] Engler H Doenlen R Riether C Engler A Niemi MB Besedovsky HO. Time-dependent alterations of peripheral immune parameters after nigrostriatal dopamine depletion in a rat model of Parkinson's disease. Brain Behav Immun. (2009) 23:518–26. 10.1016/j.bbi.2009.01.018 19486644

[173] Benner EJ Banerjee R Reynolds AD Sherman S Pisarev VM Tsiperson V. Nitrated alpha-synuclein immunity accelerates degeneration of nigral dopaminergic neurons. PLoS ONE. (2008) 3:e1376. 10.1371/journal.pone.0001376 18167537 PMC2147051

[174] Reynolds AD Stone DK Hutter JA Benner EJ Mosley RL Gendelman HE. Regulatory T cells attenuate Th17 cell-mediated nigrostriatal dopaminergic neurodegeneration in a model of Parkinson's disease. J Immunol. (2010) 184:2261–71. 10.4049/jimmunol.0901852 20118279 PMC2824790

[175] Reynolds AD Banerjee R Liu J Gendelman HE Mosley RL. Neuroprotective activities of CD4+CD25+ regulatory T cells in an animal model of Parkinson's disease. J Leukoc Biol. (2007) 82:1083–94. 10.1189/jlb.0507296 17675560

[176] Lacan G Dang H Middleton B Horwitz MA Tian J Melega WP. Bacillus Calmette-Guerin vaccine-mediated neuroprotection is associated with regulatory T-cell induction in the 1-methyl-4-phenyl-1,2,3,6-tetrahydropyridine mouse model of Parkinson's disease. J Neurosci Res. (2013) 91:1292–302. 10.1002/jnr.23253 23907992 PMC5800426

[177] Chung ES Kim H Lee G Park S Kim H Bae H. Neuro-protective effects of bee venom by suppression of neuroinflammatory responses in a mouse model of Parkinson's disease: role of regulatory T cells. Brain Behav Immun. (2012) 26:1322–30. 10.1016/j.bbi.2012.08.013 22974722

[178] Chi DS Gong L Daigneault EA Kostrzewa RM. Effects of MPTP and vitamin E treatments on immune function in mice. Int J Immunopharmacol. (1992) 14:739–46. 10.1016/0192-0561(92)90070-2 1512070

[179] Wheeler CJ Seksenyan A Koronyo Y Rentsendorj A Sarayba D Wu H. T-lymphocyte deficiency exacerbates behavioral deficits in the 6-OHDA unilateral lesion rat model for Parkinson's disease. J Neurol Neurophysiol. (2014) 5:209. 10.4172/2155-9562.1000209 25346865 PMC4207300

[180] Schetters STT Gomez-Nicola D Garcia-Vallejo JJ Van Kooyk Y. Neuroinflammation: Microglia and T Cells Get Ready to Tango. Front Immunol. (2017) 8:1905. 10.3389/fimmu.2017.01905 29422891 PMC5788906

[181] Pare A Mailhot B Levesque SA Juzwik C Ignatius Arokia Doss PM Lecuyer MA IL-1beta enables CNS access to CCR2(hi) monocytes and the generation of pathogenic cells through GM-CSF released by CNS endothelial cells. Proc Natl Acad Sci USA. (2018) 115:E1194–E1203. 10.1073/pnas.1714948115 29358392 PMC5819409

[182] Hettinger J Richards DM Hansson J Barra MM Joschko AC Krijgsveld J. Origin of monocytes and macrophages in a committed progenitor. Nat Immunol. (2013) 14:821–30. 10.1038/ni.2638 23812096

[183] Geissmann F Manz MG Jung S Sieweke MH Merad M Ley K. Development of monocytes, macrophages, and dendritic cells. Science. (2010) 327:656–61. 10.1126/science.1178331 20133564 PMC2887389

[184] Prinz M Erny D Hagemeyer N Ontogeny and homeostasis of CNS myeloid cells. Nat Immunol. (2017) 18:385–92. 10.1038/ni.3703 28323268

[185] Gomez Perdiguero E Klapproth K Schulz C Busch K Azzoni E Crozet L. Tissue-resident macrophages originate from yolk-sac-derived erythro-myeloid progenitors. Nature. (2015) 518:547–51. 10.1038/nature13989 25470051 PMC5997177

[186] Bennett FC Bennett ML Yaqoob F Mulinyawe SB Grant GA Hayden Gephart M. A combination of ontogeny and CNS environment establishes microglial identity. Neuron. (2018) 98:1170–83 e1178. 10.1016/j.neuron.2018.05.014 29861285 PMC6023731

[187] Prinz M Priller J. Tickets to the brain: role of CCR2 and CX3CR1 in myeloid cell entry in the CNS. J Neuroimmunol. (2010) 224:80–4. 10.1016/j.jneuroim.2010.05.015 20554025

[188] Saederup N Cardona AE Croft K Mizutani M Cotleur AC Tsou CL. Selective chemokine receptor usage by central nervous system myeloid cells in CCR2-red fluorescent protein knock-in mice. PLoS ONE. (2010) 5:e13693. 10.1371/journal.pone.0013693 21060874 PMC2965160

[189] Chu HX Arumugam TV Gelderblom M Magnus T Drummond GR Sobey CG. Role of CCR2 in inflammatory conditions of the central nervous system. J Cereb Blood Flow Metab. (2014) 34:1425–9. 10.1038/jcbfm.2014.120 24984897 PMC4158674

[190] Lopez-Atalaya JP Askew KE Sierra A Gomez-Nicola D. Development and maintenance of the brain's immune toolkit: Microglia and non-parenchymal brain macrophages. Dev Neurobiol. (2018) 78:561–79. 10.1002/dneu.22545 29030904 PMC6001428

[191] Schlachetzki JCM Prots I Tao J Chun HB Saijo K Gosselin D. A monocyte gene expression signature in the early clinical course of Parkinson's disease. Sci Rep. (2018) 8:10757. 10.1038/s41598-018-28986-7 30018301 PMC6050266

[192] Grozdanov V Bliederhaeuser C Ruf WP Roth V Fundel-Clemens K Zondler L. Inflammatory dysregulation of blood monocytes in Parkinson's disease patients. Acta Neuropathol. (2014) 128:651–63. 10.1007/s00401-014-1345-4 25284487 PMC4201759

[193] Cote M Poirier AA Aube B Jobin C Lacroix S Soulet D Partial depletion of the proinflammatory monocyte population is neuroprotective in the myenteric plexus but not in the basal ganglia in a MPTP mouse model of Parkinson's disease. Brain Behav Immun. (2015) 46:154–67. 10.1016/j.bbi.2015.01.009 25637482

[194] Parillaud VR Lornet G Monnet Y Privat AL Haddad AT Brochard V. Analysis of monocyte infiltration in MPTP mice reveals that microglial CX3CR1 protects against neurotoxic over-induction of monocyte-attracting CCL2 by astrocytes. J Neuroinflammation. (2017) 14:60. 10.1186/s12974-017-0830-9 28320442 PMC5359822

[195] Broz P Dixit VM. Inflammasomes: mechanism of assembly, regulation and signalling. Nat Rev Immunol. (2016) 16:407–20. 10.1038/nri.2016.58 27291964

[196] Heneka MT. Inflammasome activation and innate immunity in Alzheimer's disease. Brain Pathol. (2017) 27:220–2. 10.1111/bpa.12483 28019679 PMC8029274

[197] Inouye BM Hughes FM Jr Sexton SJ Purves JT. The emerging role of inflammasomes as central mediators in inflammatory bladder pathology. Curr Urol. (2018) 11:57–72. 10.1159/000447196 29593464 PMC5836190

[198] Elliott EI Sutterwala FS. Initiation and perpetuation of NLRP3 inflammasome activation and assembly. Immunol Rev. (2015) 265:35–52. 10.1111/imr.12286 25879282 PMC4400874

[199] Seo MJ Hong JM Kim SJ Lee SM. Genipin protects d-galactosamine and lipopolysaccharide-induced hepatic injury through suppression of the necroptosis-mediated inflammasome signaling. Eur J Pharmacol. (2017) 812:128–37. 10.1016/j.ejphar.2017.07.024 28709622

[200] Von Herrmann KM Salas LA Martinez EM Young AL Howard JM Feldman MS. NLRP3 expression in mesencephalic neurons and characterization of a rare NLRP3 polymorphism associated with decreased risk of Parkinson's disease. NPJ Parkinsons Dis. (2018) 4:24. 10.1038/s41531-018-0061-5 30131971 PMC6093937

[201] Zhang P Shao XY Qi GJ Chen Q Bu LL Chen LJ. Cdk5-dependent activation of neuronal inflammasomes in Parkinson's Disease. Mov Disord. (2016) 31:366–76. 10.1002/mds.26488 26853432

[202] Opipari A Franchi L. Role of inflammasomes in intestinal inflammation and Crohn's disease. Inflamm Bowel Dis. (2015) 21:173–81. 10.1097/MIB.0000000000000230 25517598

[203] Villumsen M Aznar S Pakkenberg B Jess T Brudek T. Inflammatory bowel disease increases the risk of Parkinson's disease: a Danish nationwide cohort study 1977-2014. Gut. (2019) 68:18–24. 10.1136/gutjnl-2017-315666 29785965

[204] Peter I Dubinsky M Bressman S Park A Lu C Chen N. Anti-tumor necrosis factor therapy and incidence of Parkinson Disease among patients with inflammatory bowel disease. JAMA Neurol. (2018) 75:939–46. 10.1001/jamaneurol.2018.0605 29710331 PMC6142934

[205] Borghammer P Is constipation in Parkinson's disease caused by gut or brain pathology? Parkinsonism Relat Disord. (2018) 55:6–7. 10.1016/j.parkreldis.2018.08.014 30181087

[206] Lionnet A Leclair-Visonneau L Neunlist M Murayama S Takao M Adler CH. Does Parkinson's disease start in the gut? Acta Neuropathol. (2018) 135:1–12. 29039141 10.1007/s00401-017-1777-8

[207] Scheperjans F Derkinderen P Borghammer P The gut and Parkinson's Disease: hype or hope? J Parkinsons Dis. (2018) 8:S31–9. 10.3233/JPD-181477 30584161 PMC6311363

[208] Park H Lee JY Shin CM Kim JM Kim TJ Kim JW. Characterization of gastrointestinal disorders in patients with parkinsonian syndromes. Parkinsonism Relat Disord. (2015) 21:455–60. 10.1016/j.parkreldis.2015.02.005 25726518

[209] Houser MC Tansey MG. The gut-brain axis: is intestinal inflammation a silent driver of Parkinson's disease pathogenesis? NPJ Parkinsons Dis. (2017) 3:3. 10.1038/s41531-016-0002-0 28649603 PMC5445611

[210] Chapelet G Leclair-Visonneau L Clairembault T Neunlist M Derkinderen P. Can the gut be the missing piece in uncovering PD pathogenesis? Parkinsonism Relat Disord. (2018). 10.1016/j.parkreldis.2018.11.014. [Epub ahead of print]. 30448099

[211] Colombo C Cosentino M Marino F Rasini E Ossola M Blandini F. Dopaminergic modulation of apoptosis in human peripheral blood mononuclear cells: possible relevance for Parkinson's disease. Ann N Y Acad Sci. (2003) 1010:679–82. 10.1196/annals.1299.124 15033811

[212] Yan Y Jiang W Liu L Wang X Ding C Tian Z. Dopamine controls systemic inflammation through inhibition of NLRP3 inflammasome. Cell. (2015) 160:62–73. 10.1016/j.cell.2014.11.047 25594175

[213] Codolo G Plotegher N Pozzobon T Brucale M Tessari I Bubacco L. Triggering of inflammasome by aggregated alpha-synuclein, an inflammatory response in synucleinopathies. PLoS ONE. (2013) 8:e55375. 10.1371/journal.pone.0055375 23383169 PMC3561263

[214] Bose A Beal MF. Mitochondrial dysfunction in Parkinson's disease. J Neurochem. (2016) 139 (Suppl. 1):216–31. 10.1111/jnc.13731 27546335

[215] Won JH Park S Hong S Son S Yu JW. Rotenone-induced impairment of mitochondrial electron transport chain confers a selective priming signal for NLRP3 inflammasome activation. J Biol Chem. (2015) 290:27425–37. 10.1074/jbc.M115.667063 26416893 PMC4646374

[216] Zhou R Yazdi AS Menu P Tschopp J. A role for mitochondria in NLRP3 inflammasome activation. Nature. (2011) 469:221–5. 10.1038/nature09663 21124315

[217] Zhou K Shi L Wang Y Chen S Zhang J. Recent Advances of the NLRP3 Inflammasome in Central Nervous System Disorders. J Immunol Res. (2016) 2016:9238290. 10.1155/2016/9238290 27652274 PMC5019917

[218] Xu LL Wu YF Yan F Li CC Dai Z You QD. 5-(3,4-Difluorophenyl)-3-(6-methylpyridin-3-yl)-1,2,4-oxadiazole (DDO-7263), a novel Nrf2 activator targeting brain tissue, protects against MPTP-induced subacute Parkinson's disease in mice by inhibiting the NLRP3 inflammasome and protects PC12 cells against oxidative stress. Free Radic Biol Med. (2019) 134:288–303. 10.1016/j.freeradbiomed.2019.01.003 30615919

[219] Qiao C Zhang LX Sun XY Ding JH Lu M Hu G. Caspase-1 deficiency alleviates dopaminergic neuronal death via inhibiting Caspase-7/AIF Pathway in MPTP/p Mouse Model of Parkinson's Disease. Mol Neurobiol. (2017) 54:4292–302. 10.1007/s12035-016-9980-5 27339879

[220] Mao Z Liu C Ji S Yang Q Ye H Han H. The NLRP3 Inflammasome is Involved in the Pathogenesis of Parkinson's Disease in rats. Neurochem Res. (2017) 42:1104–15. 10.1007/s11064-017-2185-0 28247334

[221] Sarkar S Malovic E Harishchandra DS Ghaisas S Panicker N Charli A. Mitochondrial impairment in microglia amplifies NLRP3 inflammasome proinflammatory signaling in cell culture and animal models of Parkinson's disease. NPJ Parkinsons Dis. (2017) 3:30. 10.1038/s41531-017-0032-2 29057315 PMC5645400

[222] Qiao C Zhang Q Jiang Q Zhang T Chen M Fan Y. Inhibition of the hepatic Nlrp3 protects dopaminergic neurons via attenuating systemic inflammation in a MPTP/p mouse model of Parkinson's disease. J Neuroinflammation. (2018) 15:193. 10.1186/s12974-018-1236-z 29966531 PMC6029067

[223] Harvey K Outeiro TF. The role of LRRK2 in cell signalling. Biochem Soc Trans. (2018) 47:197–207. 10.1042/BST20180464 30578345

[224] Lesage S Durr A Tazir M Lohmann E Leutenegger AL Janin S. LRRK2 G2019S as a cause of Parkinson's disease in North African Arabs. N Engl J Med. (2006) 354:422–3. 10.1056/NEJMc055540 16436781

[225] Satake W Nakabayashi Y Mizuta I Hirota Y Ito C Kubo M. Genome-wide association study identifies common variants at four loci as genetic risk factors for Parkinson's disease. Nat Genet. (2009) 41:1303–7. 10.1038/ng.485 19915576

[226] Greggio E. Role of LRRK2 kinase activity in the pathogenesis of Parkinson's disease. Biochem Soc Trans. (2012) 40:1058–62. 10.1042/BST20120054 22988865

[227] Hui KY Fernandez-Hernandez H Hu J Schaffner A Pankratz N Hsu NY. Functional variants in the LRRK2 gene confer shared effects on risk for Crohn's disease and Parkinson's disease. Sci Transl Med. (2018) 10:eaai7795. 10.1126/scitranslmed.aai7795 29321258 PMC6028002

[228] Gardet A Benita Y Li C Sands BE Ballester I Stevens C. LRRK2 is involved in the IFN-gamma response and host response to pathogens. J Immunol. (2010) 185:5577–85. 10.4049/jimmunol.1000548 20921534 PMC3156100

[229] Hakimi M Selvanantham T Swinton E Padmore RF Tong Y Kabbach G. Parkinson's disease-linked LRRK2 is expressed in circulating and tissue immune cells and upregulated following recognition of microbial structures. J Neural Transm. (2011) 118:795–808. 10.1007/s00702-011-0653-2 21552986 PMC3376651

[230] Cook DA Kannarkat GT Cintron AF Butkovich LM Fraser KB Chang J. LRRK2 levels in immune cells are increased in Parkinson's disease. NPJ Parkinsons Dis. (2017) 3:11. 10.1038/s41531-017-0010-8 28649611 PMC5459798

[231] Hill-Burns EM Factor SA Zabetian CP Thomson G Payami H. Evidence for more than one Parkinson's disease-associated variant within the HLA region. PLoS ONE. (2011) 6:e27109. 10.1371/journal.pone.0027109 22096524 PMC3212531

[232] Ahmed I Tamouza R Delord M Krishnamoorthy R Tzourio C Mulot C. Association between Parkinson's disease and the HLA-DRB1 locus. Mov Disord. (2012) 27:1104–10. 10.1002/mds.25035 22807207

[233] Kannarkat GT Cook DA Lee JK Chang J Chung J Sandy E. Common genetic variant association with Altered HLA Expression, Synergy with Pyrethroid Exposure, and Risk for Parkinson's Disease: An Observational and Case-Control Study. NPJ Parkinsons Dis. (2015) 1:15002. 10.1038/npjparkd.2015.2 27148593 PMC4853162

[234] Ness D Ren Z Gardai S Sharpnack D Johnson VJ Brennan RJ. Leucine-rich repeat kinase 2 (LRRK2)-deficient rats exhibit renal tubule injury and perturbations in metabolic and immunological homeostasis. PLoS ONE. (2013) 8:e66164. 10.1371/journal.pone.0066164 23799078 PMC3682960

[235] Daher JP Volpicelli-Daley LA Blackburn JP Moehle MS West AB. Abrogation of alpha-synuclein-mediated dopaminergic neurodegeneration in LRRK2-deficient rats. Proc Natl Acad Sci USA. (2014) 111:9289–94. 10.1073/pnas.1403215111 24927544 PMC4078806

[236] Kozina E Sadasivan S Jiao Y Dou Y Ma Z Tan H. Mutant LRRK2 mediates peripheral and central immune responses leading to neurodegeneration *in vivo*. Brain. (2018) 141:1753–69. 10.1093/brain/awy077 29800472 PMC7190032

[237] Yun SP Kam TI Panicker N Kim S Oh Y Park JS. Block of A1 astrocyte conversion by microglia is neuroprotective in models of Parkinson's disease. Nat Med. (2018) 24:931–8. 10.1038/s41591-018-0051-5 29892066 PMC6039259

[238] Kim S Moon M Park S. Exendin-4 protects dopaminergic neurons by inhibition of microglial activation and matrix metalloproteinase-3 expression in an animal model of Parkinson's disease. J Endocrinol. (2009) 202:431–9. 10.1677/JOE-09-0132 19570816

[239] Nassar NN Al-Shorbagy MY Arab HH Abdallah DM. Saxagliptin: a novel antiparkinsonian approach. Neuropharmacology. (2015) 89:308–17. 10.1016/j.neuropharm.2014.10.007 25446674

[240] Aviles-Olmos I Dickson J Kefalopoulou Z Djamshidian A Ell P Soderlund T. Exenatide and the treatment of patients with Parkinson's disease. J Clin Invest. (2013) 123:2730–6. 10.1172/JCI68295 23728174 PMC3668846

[241] Athauda D Maclagan K Skene SS Bajwa-Joseph M Letchford D Chowdhury K. Exenatide once weekly versus placebo in Parkinson's disease: a randomised, double-blind, placebo-controlled trial. Lancet. (2017) 390:1664–75. 10.1016/S0140-6736(17)31585-4 28781108 PMC5831666

[242] Athauda D Maclagan K Budnik N Zampedri L Hibbert S Aviles-Olmos I. Post hoc analysis of the Exenatide-PD trial-Factors that predict response. Eur J Neurosci. (2018) 49:410–21. 10.1111/ejn.14096 30070753

[243] Athauda D Maclagan K Budnik N Zampedri L Hibbert S Skene SS. What effects might exenatide have on non-motor symptoms in Parkinson's Disease: a post hoc analysis. J Parkinsons Dis. (2018) 8:247–58. 10.3233/JPD-181329 29843254

[244] Wu DC Jackson-Lewis V Vila M Tieu K Teismann P Vadseth C. Blockade of microglial activation is neuroprotective in the 1-methyl-4-phenyl-1,2,3,6-tetrahydropyridine mouse model of Parkinson disease. J Neurosci. (2002) 22:1763–71. 10.1523/JNEUROSCI.22-05-01763.2002 11880505 PMC6758858

[245] Investigators NN-P A pilot clinical trial of creatine and minocycline in early Parkinson disease: 18-month results. Clin Neuropharmacol. (2008) 31:141–50. 10.1097/WNF.0b013e3181342f32 18520981 PMC4372145

[246] Lazzarini M Martin S Mitkovski M Vozari RR Stuhmer W Bel ED. Doxycycline restrains glia and confers neuroprotection in a 6-OHDA Parkinson model. Glia. (2013) 61:1084–100. 10.1002/glia.22496 23595698

[247] Zhang GB Feng YH Wang PQ Song JH Wang P Wang SA. A study on the protective role of doxycycline upon dopaminergic neuron of LPS-PD rat model rat. Eur Rev Med Pharmacol Sci. (2015) 19:3468–74. 26439044

[248] Bortolanza M Nascimento GC Socias SB Ploper D Chehin RN Raisman-Vozari R. Tetracycline repurposing in neurodegeneration: focus on Parkinson's disease. J Neural Transm. (2018) 125:1403–15. 10.1007/s00702-018-1913-1 30109452

[249] Coll RC Robertson AA Chae JJ Higgins SC Munoz-Planillo R Inserra MC. A small-molecule inhibitor of the NLRP3 inflammasome for the treatment of inflammatory diseases. Nat Med. (2015) 21:248–55. 10.1038/nm.3806 25686105 PMC4392179

[250] Yin H Guo Q Li X Tang T Li C Wang H. Curcumin Suppresses IL-1beta Secretion and Prevents Inflammation through Inhibition of the NLRP3 Inflammasome. J Immunol. (2018) 200:2835–46. 10.4049/jimmunol.1701495 29549176

[251] Zou P Liu X Li G Wang Y. Resveratrol pretreatment attenuates traumatic brain injury in rats by suppressing NLRP3 inflammasome activation via SIRT1. Mol Med Rep. (2018) 17:3212–7. 29257276 10.3892/mmr.2017.8241

[252] Domiciano TP Wakita D Jones HD Crother TR Verri WA Jr Arditi M. Quercetin Inhibits Inflammasome Activation by Interfering with ASC oligomerization and prevents interleukin-1 mediated mouse vasculitis. Sci Rep. (2017) 7:41539. 10.1038/srep41539 28148962 PMC5288648

